# Activin A secretion by muscle-repairing macrophages induces heterotopic ossification in mice

**DOI:** 10.1172/JCI193797

**Published:** 2026-03-02

**Authors:** Wenqiang Yin, Kazuo Okamoto, Asuka Terashima, Warunee Pluemsakunthai, Takehito Ono, Taku Ito-Kureha, Shizuo Akira, Yoshinobu Hashizume, Roland Baron, Satoshi Ueha, Kouji Matsushima, Martin M. Matzuk, Yuji Mishina, Hiroshi Takayanagi

**Affiliations:** 1 Department of Immunology and; 2 Department of Osteoimmunology, Graduate School of Medicine and Faculty of Medicine, The University of Tokyo, Tokyo, Japan.; 3 Division of Immune Environment Dynamics, Cancer Research Institute, and; 4 Immune Network Research Unit, Institute for Frontier Science Initiative InFiniti, Kanazawa University, Kanazawa, Japan.; 5 Bone and Cartilage Regenerative Medicine, The University of Tokyo Hospital, Tokyo, Japan.; 6 Laboratory of Drug Discovery and Pharmacology, Faculty of Veterinary Medicine, Okayama University of Science, Imabari, Japan.; 7 Laboratory of Host Defense, Immunology Frontier Research Center, The University of Osaka, Osaka, Japan.; 8 RIKEN Program for Drug Discovery and Medical Technology Platforms, RIKEN TRIP Headquarters, Wako, Japan.; 9 Department of Medicine, Harvard Medical School, Massachusetts General Hospital, Endocrine Division, and Department of Oral Medicine, Infection, and Immunity, Harvard School of Dental Medicine, Boston, Massachusetts, USA.; 10 Division of Molecular Regulation of Inflammatory and Immune Diseases, Research Institute for Biomedical Sciences, Tokyo University of Science, Chiba, Japan.; 11 Center for Drug Discovery, Department of Pathology & Immunology, Baylor College of Medicine, Houston, Texas, USA.; 12 Department of Biologic and Materials Sciences & Prosthodontics, University of Michigan School of Dentistry, Ann Arbor, Michigan, USA.

**Keywords:** Bone biology, Immunology, Muscle biology, Genetic diseases, Innate immunity, Skeletal muscle

## Abstract

The immune system is not only essential for host defense, but it is also involved in tissue maintenance and disease pathogenesis. Macrophages play a key role in tissue repair, fibrosis, and tumorigenesis, but the mechanisms underlying their multifunctionality have not been fully explored. Here, we identified Mrep (Ly6C^hi^CX3CR1^lo^PDPN^+^CD9^+^) as a crucial subset of macrophages for muscle regeneration after muscle injury. Muscle regeneration required Mrep-derived activin A, which was produced via the TLR4/TIR domain–containing adapter-inducing interferon-β/TANK-binding kinase 1/interferon regulatory factor 3/7 signaling pathway in response to muscle injury. Mrep exerted pathological effects by secreting activin A in a model of genetically induced heterotopic ossification (HO), which was suppressed by TLR4 inhibition. Thus, this study elucidates the context-dependent functions of macrophages and the link between injury and HO, suggesting that Mrep is a potential therapeutic target for regenerating muscles and suppressing HO.

## Abstract 2

An activin A–producing macrophage subset aids muscle repair but can trigger abnormal extraskeletal bone formation in a rare disease context.

## Introduction

Macrophages play a key role in the innate immune system by recognizing and responding to pathogens through various scavenger, pattern recognition, and phagocytic receptors (1–3). Macrophage phagocytic activity is involved in the process of removing dead cells, which is essential for the maintenance of tissue homeostasis (4, 5). However, the discovery of disease-associated macrophages has highlighted their diverse roles in health and disease. Macrophages exacerbate tissue fibrosis in the lung and liver through the production of proinflammatory cytokines (6, 7). During tumorigenesis, macrophages facilitate the formation of the premetastatic niche and inhibit the immune response (8). Macrophages are also involved in intramuscular fat deposition (9), fibrosis (10, 11), and heterotopic ossification (HO) (12, 13), resulting in muscle degeneration under certain pathological conditions.

Despite their detrimental effects, macrophages support muscle regeneration through coordinating innate immune responses with satellite cells. Macrophage ablation has been shown to severely impair proper muscle regeneration (14–16). In particular, macrophage-derived proinflammatory cytokines such as TNF not only sustain the inflammatory response, but they also act directly on satellite cells by regulating their expression of myogenic regulatory genes (17). Studies have revealed that macrophages promote muscle regeneration by releasing glutamine (18), IGF1 (19), HGF (20), growth differentiation factor 3 (GDF3) (21), and GDF15 (22). Thus, macrophages are involved in both muscle regeneration and degeneration. However, the macrophage subsets and molecular mechanisms underlying their diverse functions remain unknown.

HO is characterized by the pathological formation of extraskeletal bone in nonosseous tissues such as muscles. Fibrodysplasia ossificans progressiva (FOP) is the most severe and debilitating form of HO, representing a uniquely catastrophic condition (23). It is caused by an activating mutation in the activin A receptor type 1 (*ACVR1*) gene that drives aberrant signaling, leading to progressive bone formation. Notably, traumatic injury triggers inflammatory episodes known as flare-ups, which dramatically accelerate HO. Nevertheless, FOP presents a life-threatening challenge with no effective treatment currently available. While macrophages are increasingly recognized as being involved in HO development in FOP (24), the precise mechanisms by which distinct macrophage subsets link traumatic injury to ectopic bone formation remain poorly understood. In the present study, through meticulous dissection of macrophage heterogeneity, we discovered a macrophage subset that plays dual roles in both physiological muscle regeneration and pathological HO in FOP. Our findings reveal the context-dependent functions of macrophages in skeletal muscle, providing crucial insights into macrophage heterogeneity and offering potential therapeutic avenues for muscle regeneration and HO suppression.

## Results

### Activin A is a muscle injury–inducible, macrophage-dependent factor that promotes myoblast proliferation.

To investigate the mechanisms of tissue repair in traumatic muscle injury, we employed a physical injury model rather than chemical induction to better mimic human trauma, in which we made unilateral incisions in the hamstrings. By analyzing the cell populations that accumulated in the injured site at different time points, we found that CD11b^+^Ly6G^–^ myeloid cells were the most abundant immune cell population in the injured site, consistently accounting for approximately 60% of CD45^+^ cells at 1 and 4 days postinjury (dpi; Supplemental Figure 1, A and B; supplemental material available online with this article; https://doi.org/10.1172/JCI193797DS1). The accumulation of the CD11b^+^Ly6G^–^ myeloid cells in injured muscles was dramatically reduced in *Ccr2*^–/–^ mice, indicating that they predominantly originate from circulating monocytes (Supplemental Figure 1, C and D). Consistent with previous studies (15, 25), most of the monocyte-derived macrophages (MDMs) at 1 dpi were proinflammatory Ly6C^hi^CX3CR1^lo^ cells, but by 4 dpi, they were markedly decreased with an increase in Ly6C^lo^CX3CR1^hi^ MDMs (Supplemental Figure 2, A and B). This phenotypic change in MDMs is essential for proper muscle regeneration (26). We next explored whether they originated from different sources. Tracking the phenotypic changes of Ly6C^hi^CX3CR1^lo^ MDMs showed their ability to transition into Ly6C^lo^CX3CR1^hi^ MDMs in muscles (Supplemental Figure 2, C–E), indicating that early Ly6C^hi^CX3CR1^lo^ MDMs contribute to the late-phase populated Ly6C^lo^CX3CR1^hi^ MDMs, as previously reported (16).

**Figure 1:**
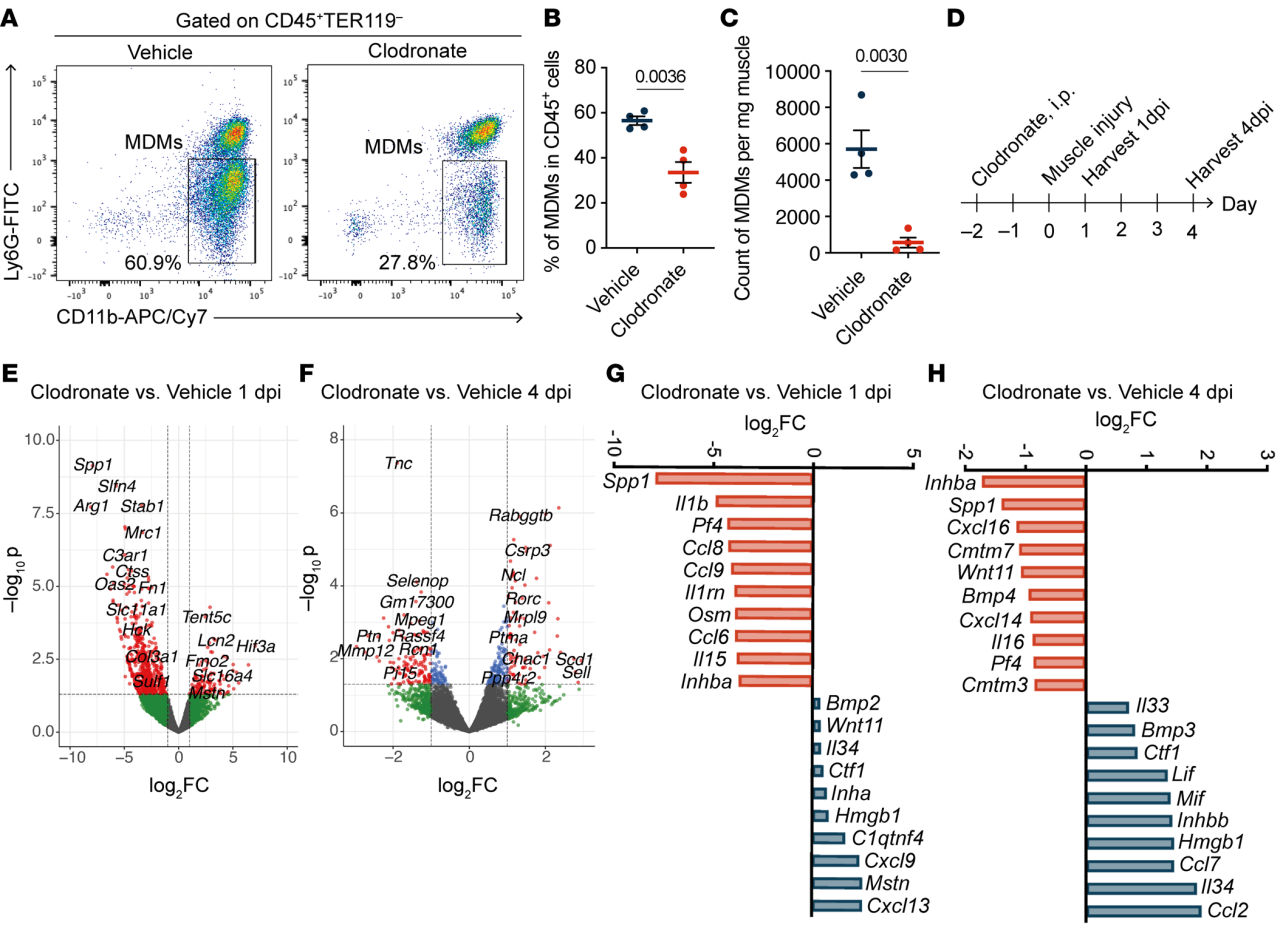
Macrophage depletion reduces muscle injury–induced activin A expression. (**A**–**C**) Representative flow cytometry plots (**A**) and statistical analysis (**B** and **C**) showing the frequency and number of neutrophils (CD11b^+^Ly6G^+^) and MDMs (CD11b^+^Ly6G^–^) in muscle tissue at 1 dpi of the mice treated with either 150 μL vehicle (*n* = 4) or clodronate (*n* = 4). (**D**) Experimental timeline of sample preparation for bulk RNA-seq. WT mice were injected intraperitoneally with 150 μL of vehicle or clodronate 2 days prior to muscle injury. Muscle injury was induced on day 0. Injured muscle samples were harvested at 1 dpi (vehicle, *n* = 2; clodronate, *n* = 3) and 4 dpi (vehicle, *n* = 2; clodronate, *n* = 2). (**E** and **F**) DEGs in muscle of clodronate-injected mice at 1 dpi (**E**) and 4 dpi (**F**) compared with vehicle-injected mice. Cutoff lines are drawn at log_2_fold change (FC) = ±1 and *P* = 0.05. Red dots represent genes with an absolute log_2_FC ≥ 1 and *P* < 0.05. Blue dots represent genes with an absolute log_2_FC < 1 and *P* < 0.05. Green dots represent genes with an absolute log_2_FC ≥ 1 and *P* > 0.05. (**G** and **H**) Top 10 DEGs encoding soluble factors (GO term: cytokine activity), ranked by FC, in muscles of clodronate-injected mice compared with vehicle-injected mice at 1 dpi (**G**) and 4 dpi (**H**). The *P* values were calculated using unpaired 2-tailed *t* test (**B** and **C**). A *P* value < 0.05 was considered significant. Data are shown as the mean ± SEM, and symbols represent individual mice (**B** and **C**).

**Figure 2:**
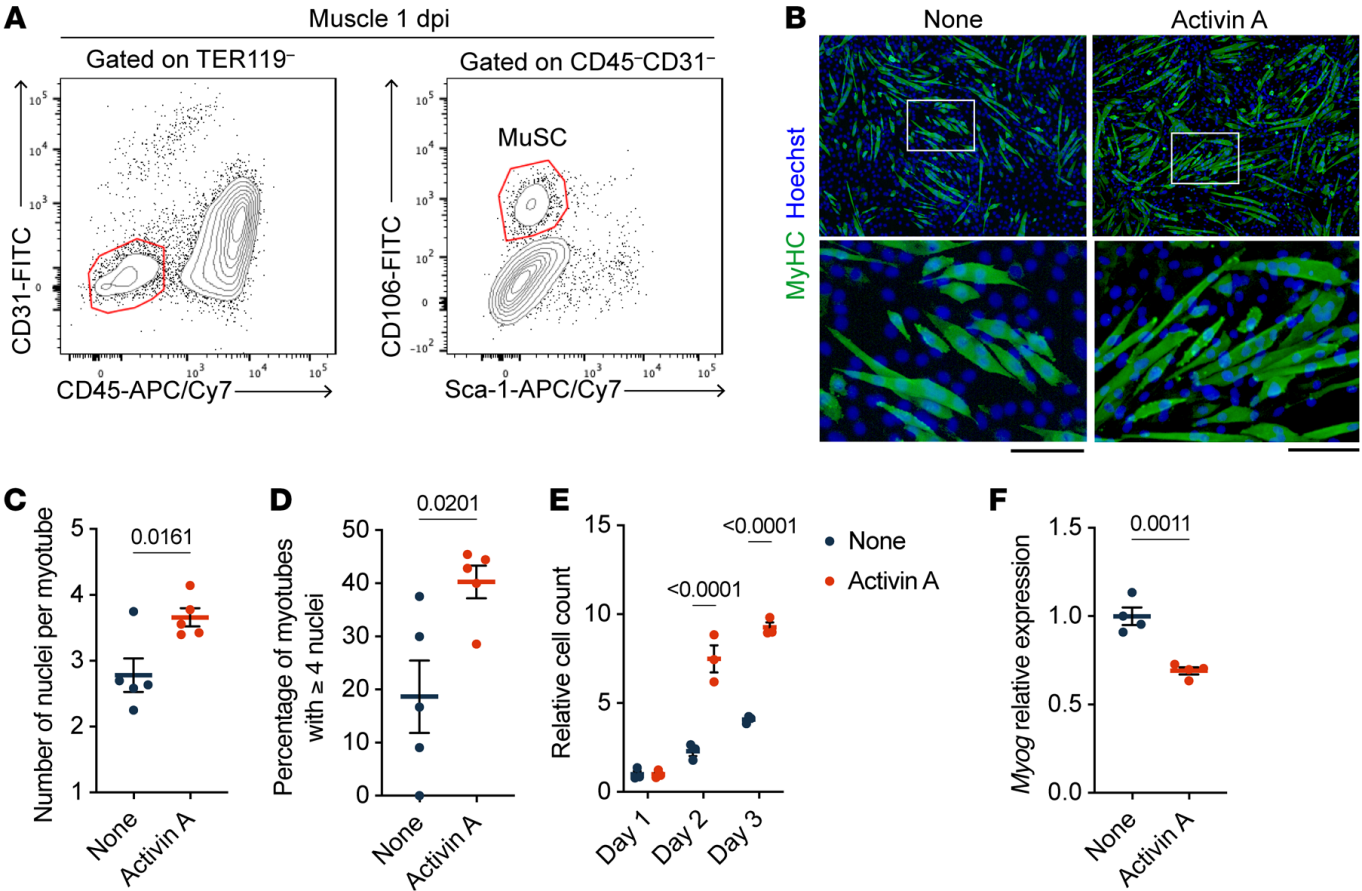
Activin A stimulates myoblast proliferation. (**A**) Representative flow cytometry plots showing the gating strategy for isolating MuSCs (TER119^–^CD45^–^CD31^–^Sca-1^–^CD106^+^) from muscles of WT mice at 1 dpi. (**B**) Representative immunofluorescence images showing MyHC^+^ myotubes (green) and nuclei (Hoechst; blue) in myoblast cultures with or without 100 ng/mL activin A treatment on day 7. MuSCs were expanded for 4 days to over 90% confluency and differentiated for 3 days. The boxed areas are shown at higher magnification (×4) in the adjacent lower panels. Scale bars: 100 μm. (**C** and **D**) Quantification of myotube fusion in **B**, showing the number of nuclei per myotube (**C**) and the percentage of myotubes with more than 4 nuclei (**D**). (**E**) Relative count of myoblasts on the indicated days. MuSCs were isolated on day 0 and then cultured for 1 to 3 days with or without 100 ng/mL activin A. (**F**) RT-qPCR analysis showing relative *Myog* mRNA expression in myoblasts treated with or without 100 ng/mL activin A for 2 days under differentiation conditions. The *P* values were calculated using unpaired 2-tailed *t* test (**C**, **D**, and **F**) or 2-way ANOVA with Bonferroni correction for multiple comparisons (**E**). A *P* value < 0.05 was considered significant. Data are shown as the mean ± SEM.

Macrophage depletion by clodronate treatment led to impaired muscle regeneration (16), although a comprehensive view of the gene expression impacted by macrophage ablation was unclear. Therefore, we performed bulk RNA-seq on the hamstring muscles from vehicle or clodronate-treated mice at 1 and 4 dpi (Figure 1, A–D). Consistent with the flow cytometry data showing that clodronate treatment reduced the number of MDMs in muscles at 1 dpi (Figure 1, A–C), the expression of macrophage function–related genes (*Arg1*, *Mrc1*, *C3ar1*, *Ccr5*, *Oas2*, *Irf7*, and *Slfn4*) was decreased in the clodronate-treated mice at 1 dpi (Figure 1E). At 4 dpi, the expression of genes involved in extracellular matrix and muscle functions (*Tnc*, *Myo1f*, *Ptn*, *Rcn1*, and *Dsel*) was much lower in clodronate-treated mice compared with vehicle-treated mice (Figure 1F). Next, we focused on the gene expression of soluble factors downregulated by clodronate treatment. At 1 dpi, the expression of proinflammatory cytokines (*Il1b* and *Il15*) and chemokines (*Ccl8*, *Ccl9*, and *Ccl6*) was markedly reduced in clodronate-treated mice (Figure 1G). Notably, *Spp1*, *Pf4*, and *Inhba* were ranked among the top 10 genes downregulated by clodronate at both 1 and 4 dpi (Figure 1, G and H), indicating a persistent rather than transient expression in MDMs after muscle injury. The contribution of *Inhba* (encoding activin A), which belongs to the TGF-β superfamily, to muscle regeneration remains controversial (27–29), and the effect of activin A on muscle satellite cells (MuSCs), the myogenic precursors, is poorly understood. We found that activin A promoted myotube formation from primary MuSCs in an in vitro culture system (Figure 2, A–D). Activin A markedly enhanced myoblast proliferation (Figure 2E) but not the expression of *Myog* (Figure 2F), which controls the terminal differentiation and fusion of myoblasts into multinucleated myotubes (30). These results indicate that activin A supports muscle regeneration by directly stimulating MuSCs proliferation without enhancing fusion.

### Ly6C^hi^PDPN^+^ MDMs produce activin A in injured skeletal muscles.

**Figure 3:**
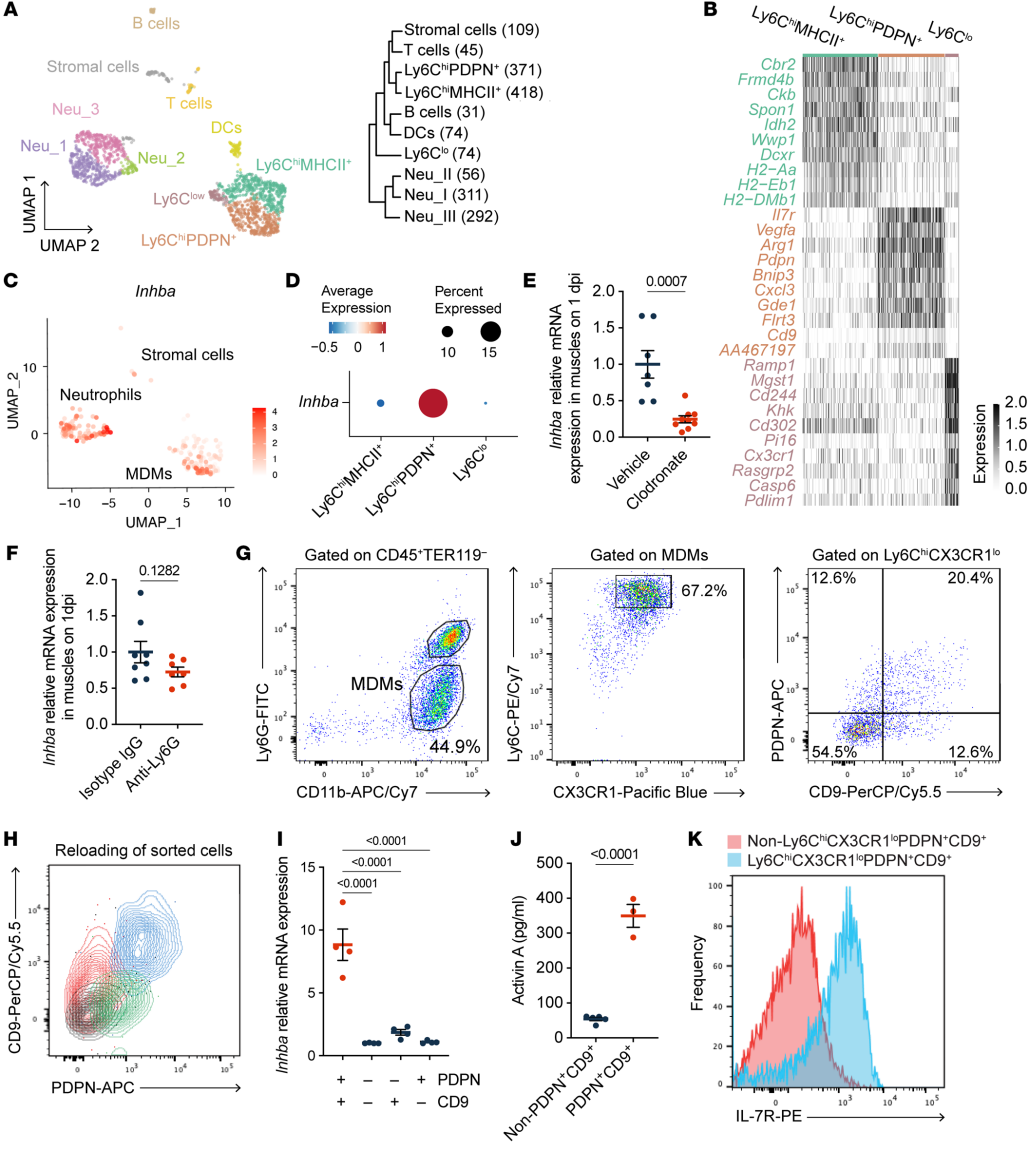
Ly6C^hi^CX3CR1^lo^PDPN^+^CD9^+^ MDMs preferentially express activin A in injured muscle. (**A**) UMAP visualization of cells isolated from muscle at 1 dpi. Phylogenetic tree showing cluster hierarchy and cell numbers (in parentheses). (**B**) Heatmap showing the top 10 DEGs by log_2_FC (adjusted *P* < 0.001) across clusters. Typical cell surface molecules were selected for naming. Expression of Ly6C is shown in Supplemental Figure 5C. (**C**) UMAP showing the expression of *Inhba* in each cluster. (**D**) Dot plot showing the relative *Inhba* expression across 3 subclusters of MDMs. (**E** and **F**) RT-qPCR results showing relative *Inhba* mRNA expression in muscles at 1 dpi from mice treated with 150 μL vehicle (*n* = 7) or clodronate (*n* = 9) (**E**) and from mice treated with 0.25 mg isotype IgG (*n* = 8) or 0.25 mg anti-Ly6G antibodies (*n* = 7) (**F**). (**G**) Gating strategy for sorting 4 subpopulations of Ly6C^hi^CX3CR1^lo^ MDMs based on the expression of PDPN and CD9. (**H**) Flow cytometric analysis confirming the purity of sorted cells. (**I**) RT-qPCR results showing relative *Inhba* expression in indicated subpopulations sorted from muscles on 1 dpi (*n* = 4 for each). The expression in the PDPN^–^CD9^–^ fraction was set to 1. (**J**) ELISA results showing the concentration of activin A in the culture supernatants of indicated MDMs sorted from muscles on 1 dpi. Supernatants were collected 48 hours after seeding. (**K**) Flow cytometric histogram showing IL-7R expression in indicated MDMs at 1 dpi. The *P* values were calculated using unpaired 2-tailed *t* test (**D**, **E**, and **J**) or 1-way ANOVA with Tukey’s multiple-comparison test (**I**). A *P* value < 0.05 was considered significant. Data are shown as the mean ± SEM, and symbols represent individual mice (**D**, **E**, **I**, and **J**).

Upon muscle injury, MDMs produced activin A (Supplemental Figure 2, F and G). To explore whether there are specific subsets of activin A–producing MDMs, we conducted scRNA-seq using cells isolated from the muscles at 1 dpi (Supplemental Figure 3A). The unbiased clustering analysis categorized 1,781 cells into 10 clusters based on the global gene expression patterns (Figure 3A, Supplemental Figure 3, B–I, and Supplemental Figure 4). Among the MDMs, 3 subclusters were identified as Ly6C^hi^MHC class II (MHCII)^+^, Ly6C^hi^PDPN^+^, and Ly6C^low^ subsets (Figure 3, A and B). Activin A was detected mainly in Ly6C^hi^PDPN^+^ MDMs, neutrophils, and, to a much lesser extent, stromal cells (Figure 3, C and D). Macrophage depletion resulted in a significant decrease in activin A mRNA expression in muscle at 1 dpi (Figure 3E), whereas neutrophil depletion did not affect the activin A expression (Figure 3F and Supplemental Figure 5, A and B), indicating that in the injured muscle the Ly6C^hi^PDPN^+^ MDMs contribute more to activin A production than the neutrophils.

scRNA-seq data revealed that both Ly6C^hi^MHCII^+^ and Ly6C^hi^PDPN^+^ subsets were skewed toward a proinflammatory phenotype, with increased expression of a panel of M1 macrophage–related genes compared with the Ly6C^lo^ subset (Supplemental Figure 5C). Each cluster displayed different expression patterns of M2 macrophage–related genes, reflecting the diversity of macrophages (Supplemental Figure 5D). In addition, the Ly6C^hi^MHCII^+^ subset expressed genes associated with phagocytosis, including *Mrc1* (mannose receptor 1), *C1qa*, and *C1qc* (Supplemental Figure 5E). Creatine kinase B, a gene involved in limiting myoblast fusion (31), was expressed in Ly6C^hi^MHCII^+^ and Ly6C^lo^ subsets, but not in the Ly6C^hi^PDPN^+^ subset (Supplemental Figure 5F). Furthermore, several cytokines that promote myogenic differentiation and muscle regeneration (such as *Cxcl10*, *Tnf*, and *Il15*) were mainly expressed in the Ly6C^hi^PDPN^+^ subcluster; they were also detected at lower levels in the Ly6C^hi^MHCII^+^ subcluster (Supplemental Figure 5G) (32–34). These findings suggest that Ly6C^hi^PDPN^+^ MDMs may have a distinct role during muscle regeneration.

### Activin A–producing macrophage subset is required for muscle regeneration.

**Figure 4:**
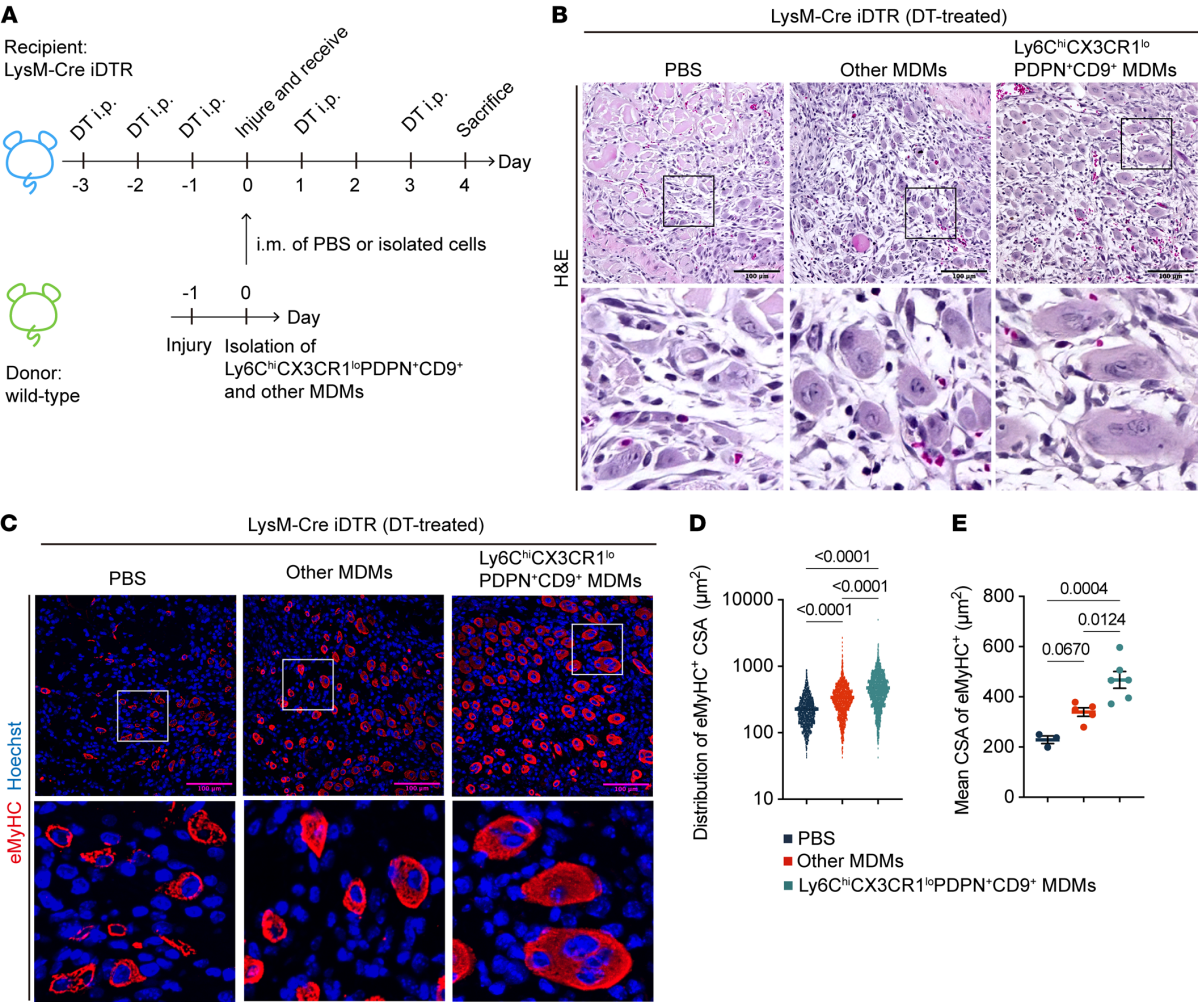
Transfer of Ly6C^hi^CX3CR1^lo^PDPN^+^CD9^+^ MDMs (Mrep) improves impaired muscle regeneration in macrophage-depleted mice. (**A**) Experimental timeline of the transfer experiment. DT (400 ng/mouse) was administered intraperitoneally on the indicated days to LysM-Cre iDTR mice to induce macrophage depletion. Ly6C^hi^CX3CR1^lo^PDPN^+^CD9^+^ MDMs and other MDMs (the remaining MDM population) were isolated from muscle tissues of WT mice at 1 dpi. The isolated cells, suspended in 10 μL PBS or the same volume of PBS alone, were then injected intramuscularly into the recipient mice. (**B** and **C**) Representative images of H&E (**B**) and immunofluorescence staining (**C**) of muscle sections from DT-treated LysM-Cre iDTR mice injected intramuscularly with PBS (*n* = 3), other MDMs (*n* = 5), or Ly6C^hi^CX3CR1^lo^PDPN^+^CD9^+^ MDMs (*n* = 6) at 4 dpi. Muscle sections were stained with anti-mouse eMyHC (red) to mark regenerating muscle fibers and Hoechst (blue) to label nuclei. The boxed areas in the main images are shown at higher magnification (×4) in the adjacent lower panels. H&E staining and corresponding immunofluorescence images represent the same field of view. Scale bars: 100 μm. (**D** and **E**) Quantification of muscle regeneration showing the distribution of the CSA of eMyHC^+^ fiber (**D**) and the mean CSA of eMyHC^+^ fibers in an individual mouse (**E**). The *P* values were calculated using 1-way ANOVA with Tukey’s multiple-comparison test. A *P* value < 0.05 was considered significant. Data are shown as the mean ± SEM, and symbols represent individual fibers (**D**) or individual mice (**E**).

Even with subclustering analysis of the MDM compartment, we found that *Inhba*-expressing subclusters were characterized by preferential expression of genes such as *Pdpn*, *Cd9*, and *Il7r*, specifically found in the Ly6C^hi^PDPN^+^ subset (Supplemental Figure 5, H–K). Thus, to explore the role of the activin A–producing MDM subset in muscle regeneration in vivo, we isolated the PDPN^+^CD9^+^ fraction from Ly6C^hi^CX3CR1^lo^ MDMs, corresponding to the Ly6C^hi^PDPN^+^ subset defined in scRNA-seq (Figure 3, G and H). We confirmed that Ly6C^hi^CX3CR1^lo^PDPN^+^CD9^+^ MDMs expressed higher levels of activin A and IL-7R compared with other MDMs (Figure 3, I–K). We found that Ly6C^hi^CX3CR1^lo^PDPN^+^CD9^+^ MDMs present on day 1 after injury underwent phenotypic changes into Ly6C^lo^CX3CR1^hi^ cells by day 4 (Supplemental Figure 6A), indicating that Ly6C^hi^CX3CR1^lo^PDPN^+^CD9^+^ MDMs are mainly active during the acute postinjury phase. LysM-Cre iDTR mice enable macrophages to be depleted upon diphtheria toxin (DT) treatment (Figure 4A and Supplemental Figure 6B). The impaired muscle regeneration in macrophage-depleted mice was improved by transferring Ly6C^hi^CX3CR1^lo^PDPN^+^CD9^+^ MDMs into the injured mice, as evidenced by a marked increase in the cross-sectional area (CSA) of regenerating fibers at 4 dpi (Figure 4, B–E). By contrast, transferring other Ly6C^hi^CX3CR1^lo^ macrophage populations had no effect. Taken together, our results show that the Ly6C^hi^CX3CR1^lo^PDPN^+^CD9^+^ MDMs are required for muscle regeneration and were thus given the acronym Mrep for macrophage directing tissue repair.

**Figure 5:**
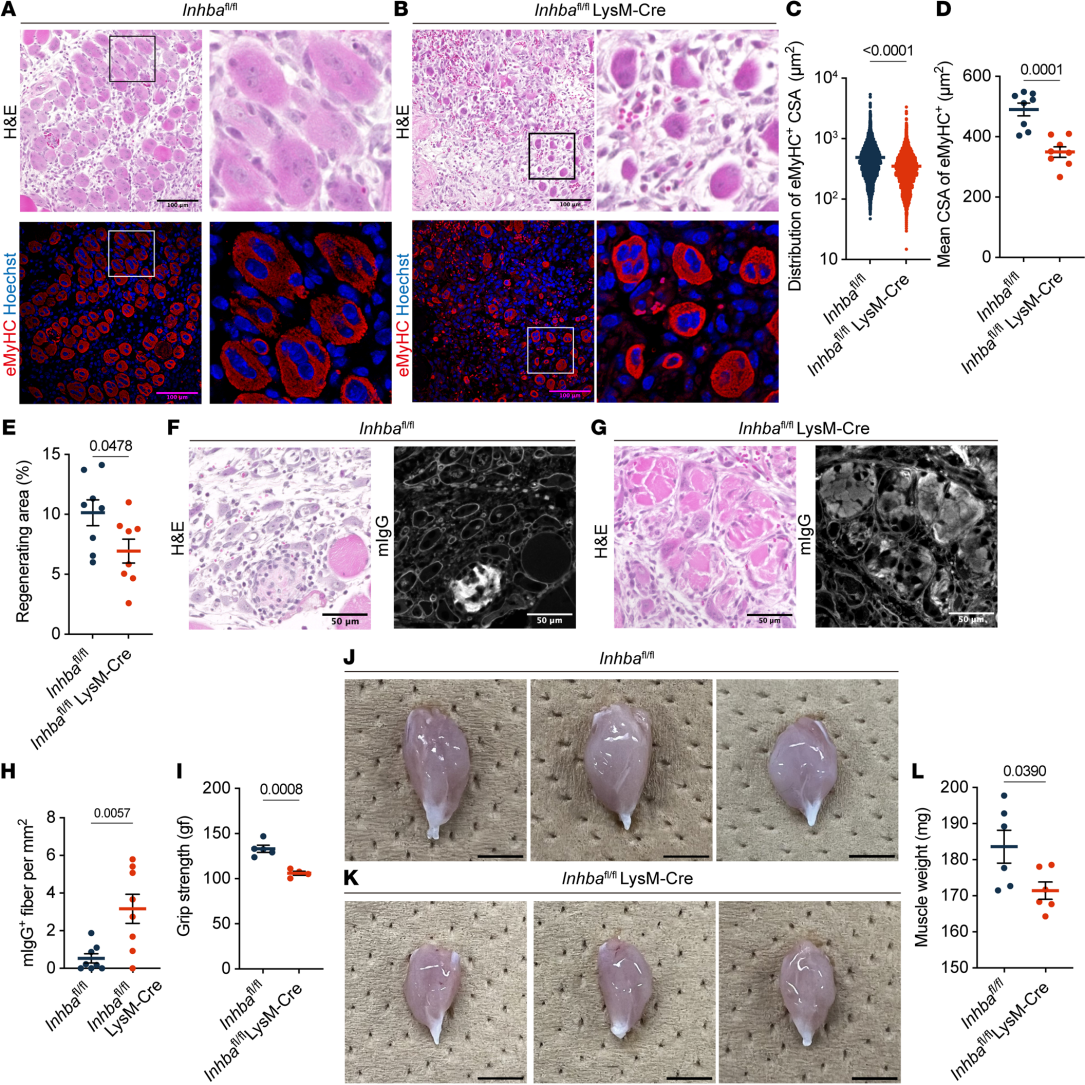
Mrep-derived activin A is essential for proper muscle regeneration. (**A** and **B**) Representative images of H&E and immunofluorescence staining of muscle sections from *Inhba*^fl/fl^ mice (*n* = 8) and *Inhba*^fl/fl^ LysM-Cre mice (*n* = 8) at 4 dpi. In immunofluorescence staining, sections were stained for anti-mouse eMyHC (red) to indicate regenerating fibers and Hoechst (blue) to indicate nuclei. The boxed areas are magnified (×4) in the panels to the right. H&E staining and corresponding immunofluorescence images represent the same field of view. Scale bars: 100 μm. (**C**–**E**) Quantification of regeneration showing the distribution of the CSA of eMyHC^+^ fiber (**C**), the mean CSA of eMyHC^+^ fibers in an individual mouse (**D**), and the percentage of regeneration area (calculated as total eMyHC^+^ area/injured area) (**E**). (**F** and **G**) Representative images of H&E and immunofluorescence staining of muscle sections from *Inhba*^fl/fl^ mice (*n* = 8) and *Inhba*^fl/fl^ LysM-Cre mice (*n* = 8) at 4 dpi. In immunofluorescence staining, sections were stained for mIgG (white) to indicate necrotic fibers. H&E staining and corresponding immunofluorescence images represent the same field of view. Scale bars: 50 μm. (**H**) The density of mIgG^+^ fibers (calculated as the number mIgG^+^ fibers/injured area). (**I**) Grip strength of the forelimb in *Inhba*^fl/fl^ and LysM-Cre *Inhba*^fl/fl^ mice at 7 dpi. gf, gram force. (**J** and **K**) Photographs of gastrocnemius of the *Inhba*^fl/fl^ (**J**) and LysM-Cre *Inhba*^fl/fl^ (**K**) mice at 14 dpi. Scale bars: 5 mm. (**L**) Mass of gastrocnemius of *Inhba*^fl/fl^ and LysM-Cre *Inhba*^fl/fl^ mice at 14 dpi. The *P* values were calculated using unpaired 2-tailed *t* test. A *P* value < 0.05 was considered significant. Data are shown as the mean ± SEM, and symbols represent individual fibers (**C**) or individual mice (**D**, **E**, **H**, **I**, and **L**).

We next investigated the role of MDM-derived activin A in muscle regeneration using macrophage-specific activin A–deficient (*Inhba*^fl/fl^ LysM-Cre) mice, in which the MDM infiltration remained unchanged (Supplemental Figure 6, C and D). At 4 dpi, regenerating myofibers were densely aligned in *Inhba*^fl/fl^ mice but sparsely distributed in *Inhba*^fl/fl^ LysM-Cre mice (Figure 5, A and B). The CSA of regenerating fibers was significantly reduced in *Inhba*^fl/fl^ LysM-Cre mice (Figure 5, C and D), along with a decrease in the regeneration area ratio (Figure 5E), indicating that the loss of activin A led to impaired muscle regeneration. Additionally, the removal of necrotic fibers was delayed in *Inhba*^fl/fl^ LysM-Cre mice (Figure 5, F–H). Functionally, at 7 dpi, the grip strength was reduced in *Inhba*^fl/fl^ LysM-Cre mice (Figure 5I). Subsequently, at 21 dpi, the injured muscle weight was lower in *Inhba*^fl/fl^ LysM-Cre mice (Figure 5, J–L). To further validate these observations, we performed adoptive transfer of WT or activin A–deficient Ly6C^hi^CX3CR1^lo^PDPN^+^CD9^+^ MDMs into macrophage-depleted mice (LysM-Cre iDTR). Transfer of activin A–deficient Ly6C^hi^CX3CR1^lo^PDPN^+^CD9^+^ MDMs led to a significant impairment of muscle regeneration compared with that of the WT group (Supplemental Figure 6, E–H), clearly indicating that macrophage-derived activin A is essential for skeletal muscle repair.

Notably, we identified a subcluster that preferentially expressed both activin A and markers used to identify Mrep (*Pdpn*, *Cd9*, and *Il7r*) in cardiotoxin-induced muscle injury tissues (Supplemental Figure 7, A–D). Also, scRNA-seq of human noninjured muscle samples obtained from surgically discarded tissue of healthy donors (35) identified a macrophage subset (cluster 1) that expressed activin A (Supplemental Figure 7, E–H), suggesting the possibility that this population contributes to repair processes following injury in humans. However, the scRNA-seq datasets from skeletal muscle of a Duchenne muscular dystrophy mouse model (*mdx^5cv^*) (36) and from hind limb ischemia (37) showed minimal or no activin A expression in macrophage clusters (Supplemental Figure 7, I and J), suggesting that activin A induction in macrophages is not a general feature of chronic muscle pathology, but rather a more specific response to acute muscle injury.

### The TLR4/TIR domain–containing adapter-inducing interferon-β–mediated signaling pathway regulates activin A expression in Mrep for proper muscle regeneration.

**Figure 6:**
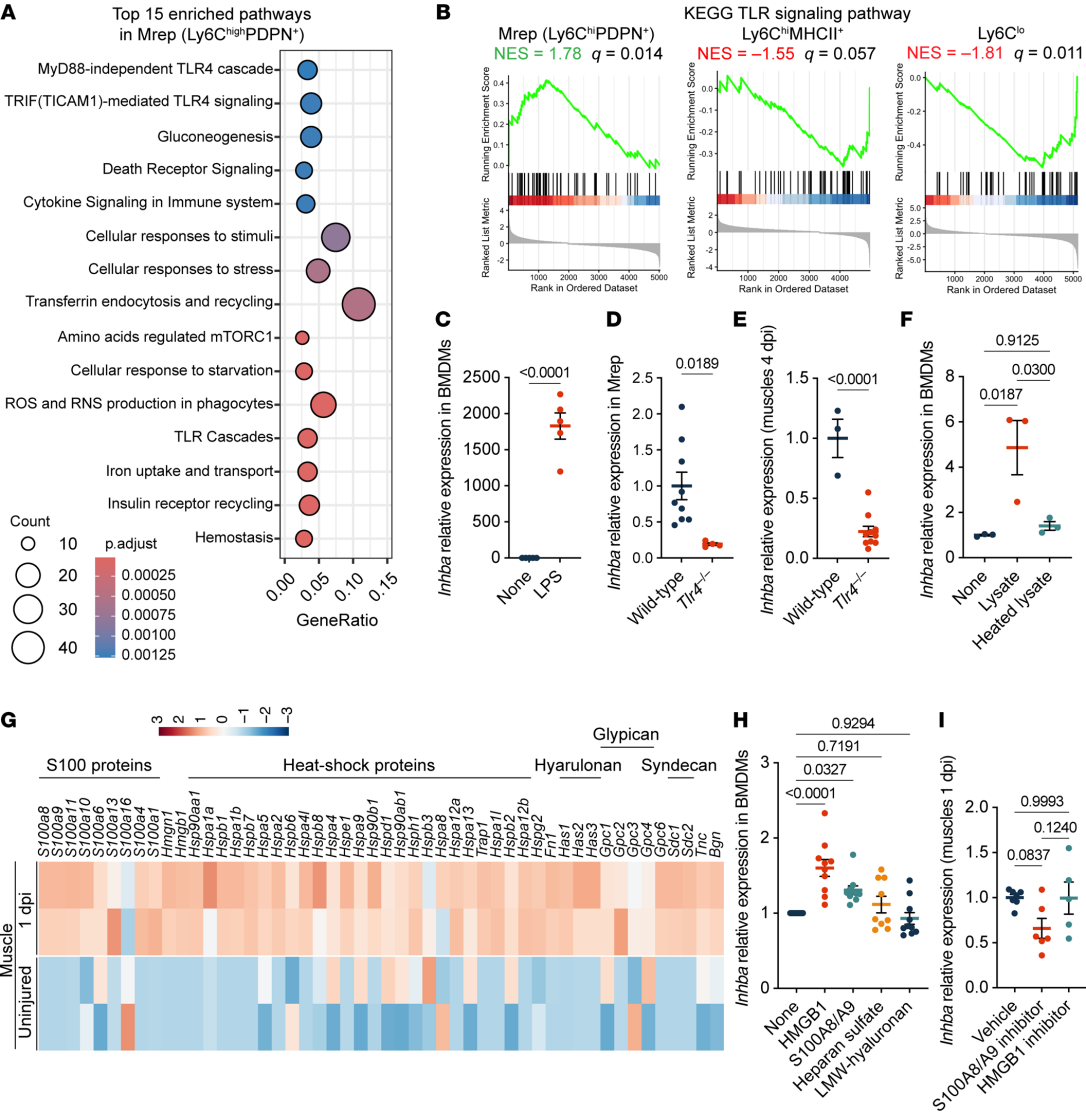
TLR4 activation by DAMPs induces activin A expression in Mrep. (**A**) Dot plot showing the top 15 enriched pathways in Mrep (Ly6C^hi^PDPN^+^ subcluster). (**B**) GSEA plots of the KEGG TLR signaling pathway in 3 MDM subclusters. NES, normalized enrichment score. (**C**) RT-qPCR results showing the relative *Inhba* expression in BMDMs stimulated with 100 ng/mL LPS for 1 day. BMDMs were derived from different mice. *n* = 5 for each group. (**D** and **E**) RT-qPCR results showing the relative *Inhba* expression in the Mrep sorted from WT (*n* = 9) and *Tlr4*^–/–^ (*n* = 4) mice at 1 dpi (**D**) and muscles from WT (*n* = 3) and *Tlr4*^–/–^ (*n* = 10) mice at 4 dpi (**E**). (**F**) RT-qPCR results showing the relative *Inhba* expression in BMDMs stimulated with muscle lysates and heated muscle lysates for 1 day. To inactivate protein, muscle lysates were subjected to 95°C for 5 minutes. BMDMs and muscle lysates were from different mice (*n* = 3). (**G**) Heatmap showing the relative expression of genes encoding DAMPs in the uninjured muscles and muscles at 1 dpi (*n* = 2 for each). (**H**) RT-qPCR results showing the relative *Inhba* mRNA expression in BMDMs treated with 1 μg/mL of indicated DAMPs for 24 hours. (**I**) RT-qPCR results showing the relative *Inhba* mRNA expression in muscles at 1 dpi of the mice injected with vehicle (*n* = 7), 10 mg/kg S100A8/A9 inhibitor (paquinimod) (*n* = 6), and HMGB1 inhibitor (glycyrrhizin) (*n* = 5). The *P* values were calculated using unpaired 2-tailed *t* test (**C**–**E**) and 1-way ANOVA with Tukey’s multiple-comparison test (**F**, **H**, and **I**). A *P* value or *q* value < 0.05 was considered significant. Data are shown as the mean ± SEM, and symbols represent individual mice.

How does Mrep produce activin A upon muscle injury? Enriched pathway analysis and GSEA indicated that the TIR domain–containing adapter-inducing interferon-β*–*mediated (TRIF-mediated) TLR4 signaling pathway and the TLR signaling pathway were enhanced in Mrep (Figure 6, A and B). Stimulation with LPS robustly increased activin A expression in BMDMs (Figure 6C). Furthermore, activin A expression in Mrep at 1 dpi, as well as in injured muscles at 4 dpi, in *Tlr4*^–/–^ mice was significantly decreased compared with that of WT mice (Figure 6, D and E), indicating that TLR4-mediated signaling is crucial for regulating activin A expression. To investigate the upstream activators of TLR4, muscle lysates from 1 dpi, which contain endogenous damage-associated molecular patterns (DAMPs), were tested in vitro. Muscle lysates induced activin A expression in BMDMs, while heated muscle lysates, in which proteins were inactivated, failed to induce activin A expression (Figure 6F), implying that protein DAMPs might be the key drivers of TLR4-mediated activin A expression. RNA-seq analysis at 1 dpi revealed an upregulation of several DAMPs, including heat shock proteins, S100 proteins, heparan sulfate proteoglycans (glypicans and syndecans), and high mobility group box1 (HMGB1) (Figure 6G). We found that HMGB1 and S100A8/A9 induced activin A expression in BMDMs in vitro (Figure 6H). However, neither HMGB1 inhibitor nor S100A8/A9 inhibitor reduced activin A expression in muscle at 1 dpi in vivo (Figure 6I), suggesting that multiple DAMPs, rather than a single DAMP, likely contribute to activin A production following muscle injury.

**Figure 7:**
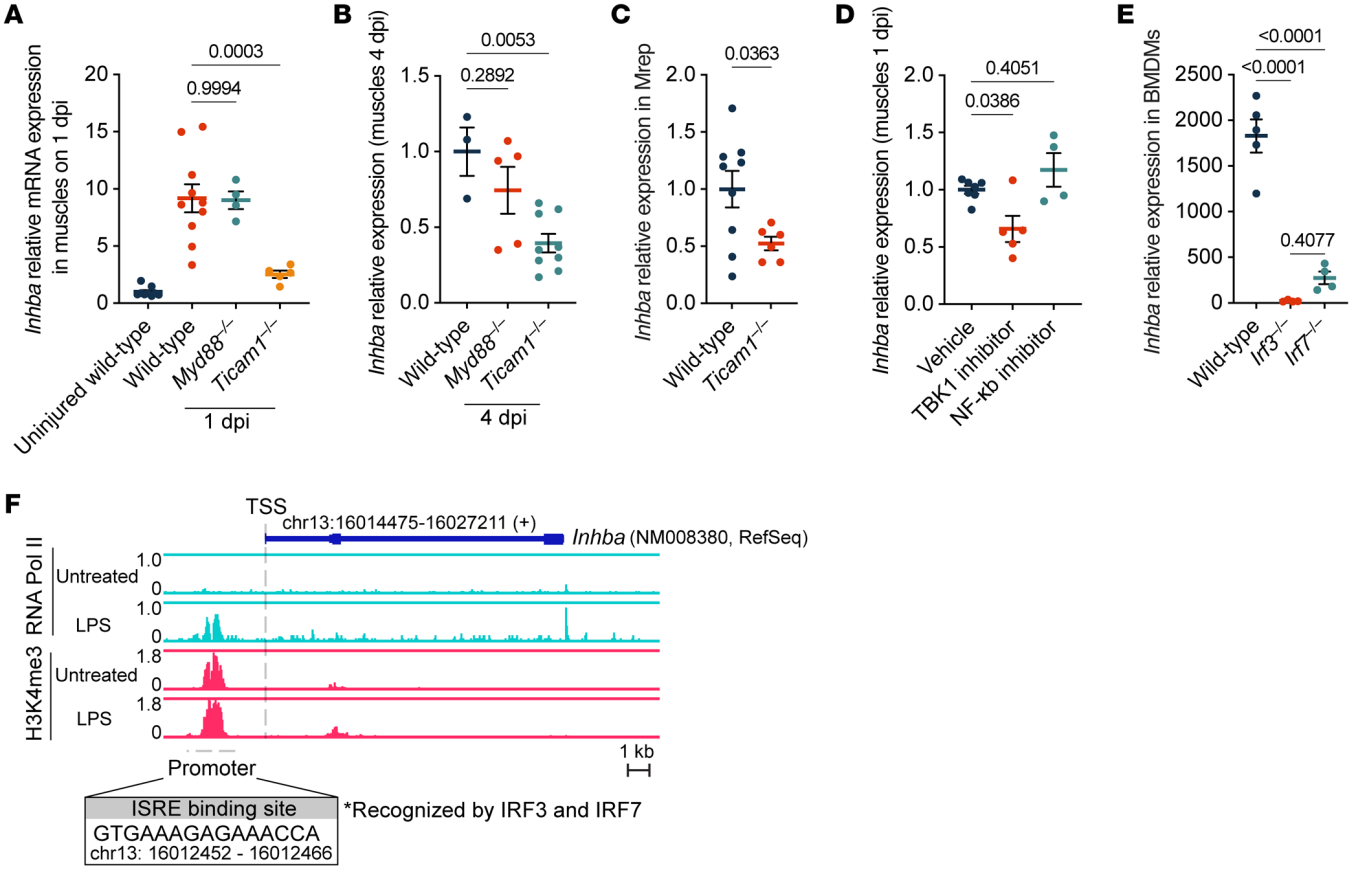
Activin A expression in Mrep is induced via the TRIF/TBK1/IRF3/7 signaling pathway. (**A** and **B**) RT-qPCR results showing the relative *Inhba* expression in muscles of indicated mice at 1 dpi (**A**) and 4 dpi (**B**). Uninjured (*n* = 9); 1 dpi: WT (*n* = 6), *Myd88*^–/–^ (*n* = 4), *Ticam1*^–/–^ (*n* = 5); 4 dpi: WT (*n* = 3), *Myd88*^–/–^ (*n* = 5), *Ticam1*^–/–^ (*n* = 9). (**C**) RT-qPCR results showing relative *Inhba* expression in the Mrep cells sorted from WT (*n* = 9) and *Ticam1*^–/–^ (*n* = 6) mice at 1 dpi. (**D**) RT-qPCR showing relative *Inhba* mRNA expression in muscles at 1 dpi in WT mice treated with vehicle (*n* = 7), TBK1 inhibitor (*n* = 5), or NF-κB inhibitor (*n* = 4). (**E**) RT-qPCR results showing relative *Inhba* mRNA expression in BMDMs derived from WT (*n* = 5), *Irf3^–/–^* (*n* = 4), or *Irf7^–/–^* mice (*n* = 4) stimulated with LPS for 1 day. (**F**) ChIP-seq (GEO GSE38377) showing RNA polymerase II (Pol II) and H3K4me3 occupancy in untreated and LPS-stimulated BMDMs at 24 hours. The *Inhba* gene is shown as a blue line with 3 exons indicated by blue boxes. Upon LPS treatment, a prominent RNA Pol II peak appeared approximately 2 kb upstream of the transcription start site (TSS), indicating the promoter. H3K4me3 enrichment in the same region indicates active chromatin. The promoter contains an interferon-stimulated responsive element (ISRE) motif recognized by IRF3 and IRF7, with its sequence and location shown in the inset. The *P* values were calculated using unpaired 2-tailed *t* test (**C**) and 1-way ANOVA with Tukey’s multiple-comparison test (**A**, **B**, **D**, and **E**). A *P* value < 0.05 was considered significant. Data are shown as the mean ± SEM, and symbols represent individual mice (**A**–**E**).

**Figure 8:**
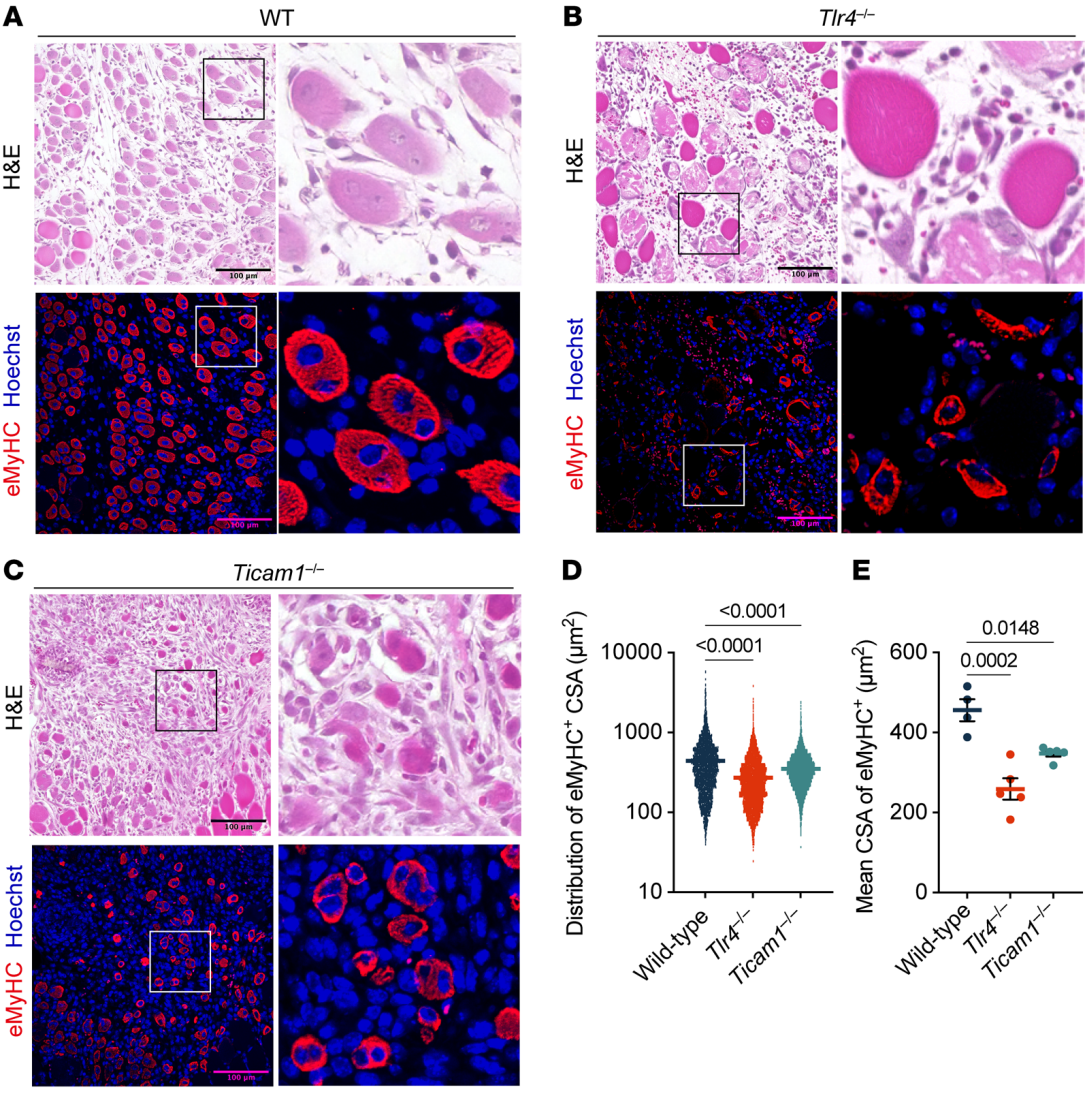
TLR4/TRIF-mediated signaling is required for proper muscle regeneration. (**A**–**C**) H&E and immunofluorescence staining of muscle sections from WT (**A**; *n* = 4), *Tlr4*^–/–^ (**B**; *n* = 5), and *Ticam1*^–/–^ mice (**C**; *n* = 5) at 4 dpi. Immunofluorescence staining showing eMyHC-labeled (red) regenerating muscle fibers and Hoechst-stained (blue) nuclei. The boxed areas are shown at higher magnification (×4) in the panels to the right. H&E staining and the corresponding immunofluorescence images represent the same field. Scale bars: 100 μm. (**D** and **E**) Quantification of regeneration showing the distribution of eMyHC^+^ CSA (**D**) and the mean CSA of eMyHC^+^ fibers (**E**). The *P* values were calculated using 1-way ANOVA with Tukey’s multiple-comparison test. A *P* value < 0.05 was considered significant. Data are shown as the mean ± SEM, and symbols represent individual fibers (**D**) or individual mice (**E**).

TLR4 activates downstream signaling pathways through myeloid differentiation primary response 88 (MyD88) and TRIF (encoded by *Ticam1*). Activin A expression was significantly suppressed in muscle at 1 and 4 dpi in TRIF-deficient mice, but not in MyD88-deficient mice (Figure 7, A and B). Furthermore, Mrep from TRIF-deficient mice decreased activin A expression (Figure 7C) without affecting Mrep frequency or number (Supplemental Figure 8, A–D), indicating that activin A is downstream of TLR4/TRIF–mediated signaling. Downstream of TLR4, TRIF activates NF-κB and TANK-binding kinase 1 (TBK1), which phosphorylates the transcriptional factors interferon regulatory factor 3 (IRF3) and IRF7 (38). We found that a TBK1 inhibitor, but not an NF-κB inhibitor, significantly inhibited activin A expression in muscles at 1 dpi (Figure 7D). Furthermore, the increased expression of activin A elicited by LPS was diminished in BMDMs derived from *Irf3^–/–^* and *Irf7^–/–^* mice (Figure 7E). ChIP-seq analysis revealed that the DNA-binding sequence for IRF3 and IRF7, the interferon-stimulated responsive element motif, is present in the promoter region of the *Inhba* gene (Figure 7F). Of note, mice deficient in TLR4 and TRIF exhibited severely impaired muscle regeneration, as evidenced by a decreased CSA of regenerating fibers (Figure 8, A–E), indicating the crucial role of the TLR4/TRIF/activin A axis in proper muscle regeneration. Taken together, Mrep contributes critically to adequate muscle regeneration by producing activin A in a TLR4/TRIF/IRF3/7 pathway–dependent manner in response to muscle injury.

### Mrep-derived activin A drives muscle injury–induced HO under conditions of aberrant ACVR1 activation.

**Figure 9:**
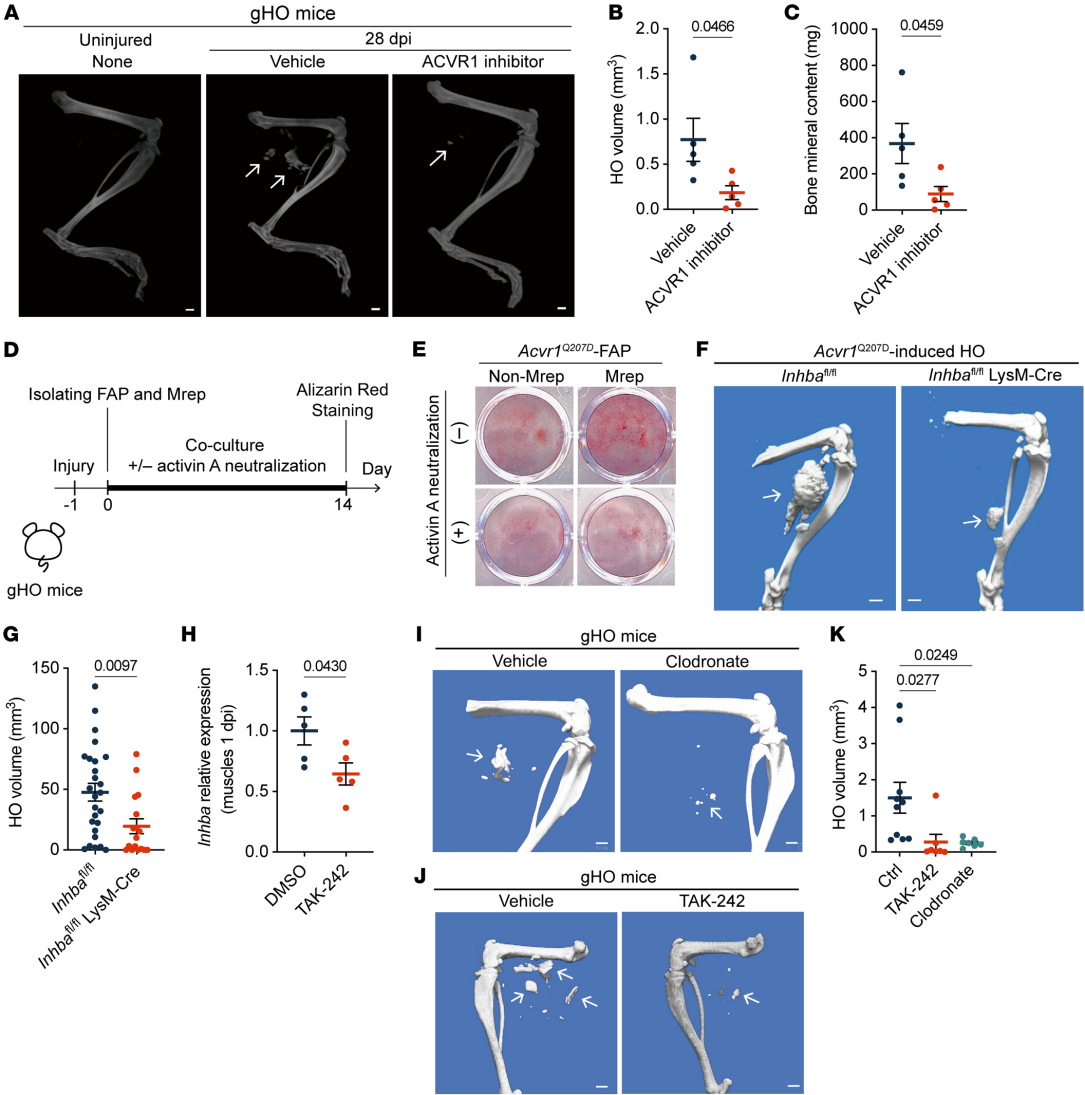
Mrep drives HO by stimulating FAPs via activin A under conditions of aberrant ACVR1 activation. (**A**) Representative microCT images of the hind limbs of uninjured (*n* = 3) and injured gHO mice treated with vehicle (*n* = 4) or ACVR1 kinase activity inhibitor (*n* = 5) at 28 dpi. White arrows indicate HO. Scale bars: 1 mm. (**B** and **C**) Quantification showing HO volume (**B**) and bone mineral content (**C**) in the gHO mice treated with vehicle or ACVR1 inhibitor. (**D**) Experimental timeline for the coculture of FAPs with Mrep. FAPs, non-FAPs, Mrep, and other cells (the remaining CD45^+^ cells) were sorted from muscles of gHO mice at 1 dpi. Anti–activin A antibody (1 mg/mL) was used to neutralize activin A. (**E**) Alizarin red S staining of the coculture experiment in **D**. Representative data from 3 independent experiments are shown. (**F** and **G**) Representative microCT images (**F**) and quantification (**G**) of HO in *Acvr1*^Q207D^-induced HO of *Inhba*^fl/fl^ mice (*n* = 26) and *Inhba*^fl/fl^ LysM-Cre mice (*n* = 17) at 28 dpi. White arrows indicate HO. Scale bars: 1 mm. (**H**) RT-qPCR results showing the relative *Inhba* expression in the muscles at 1 dpi from the mice treated with DMSO (*n* = 5) or TAK-242 (*n* = 5). (**I**–**K**) Representative microCT images (**I** and **J**) and quantification (**K**) of HO in gHO mice treated with vehicle (*n* = 10), TAK-242 (*n* = 7), and clodronate (*n* = 7) at 28 dpi. White arrows indicate HO. Scale bars: 1 mm. The *P* values were calculated using unpaired 2-tailed *t* test (**B**, **C**, **G**, and **H**) and 1-way ANOVA with Tukey’s multiple-comparison test (**K**). A *P* value < 0.05 was considered significant. Data are shown as the mean ± SEM, and symbols represent individual mice.

Although skeletal muscle regeneration generally proceeds normally and leads to functional recovery, HO can occur as a subsequent event after muscle injury under certain pathological conditions. In particular, activin A contributes to the pathogenesis of FOP via its interaction with mutant ACVR1 (39, 40). Activin A does not signal through WT ACVR1, but aberrantly activates mutated ACVR1 in patients with FOP, thereby contributing to HO following muscle injury. The findings on the property of Mrep to produce activin A in response to traumatic muscle injury prompted us to investigate a possible detrimental role for Mrep in HO in FOP. We established a genetically induced HO mouse model using *Acvr1*^Q207D^ CreER mice (hereafter called gHO mice) (Supplemental Figure 9, A and B). The expression of *Inhba* upon muscle injury was comparable in the gHO mice and WT mice (Supplemental Figure 9, C and D). HO was detected in the injured sites of the gHO mice, but not in uninjured gHO mice (Figure 9, A–C, and Supplemental Figure 9E). The muscle injury–induced HO was significantly suppressed by daily administration of LDN-193189, a specific inhibitor of type I bone morphogenetic protein (BMP) receptors with selectivity for ACVR1 (Figure 9, A–C), indicating that the HO in this model depends on ACVR1-mediated signaling.

HO originates from tissue-resident fibro/adipogenic progenitors (FAPs), characterized as CD45^–^TER119^–^CD31^–^PDGFRα^+^Sca-1^+^ cells (41–43). FAPs derived from *Acvr1*^Q207D^ mice underwent robust osteogenic differentiation in response to activin A treatment, whereas non-FAP populations, including MuSCs, failed to do so (Supplemental Figure 9, F and G). Furthermore, the osteogenic differentiation of ACVR1 Q207D mutant–expressing FAPs was induced by coculture with Mrep but not with other CD45^+^ cells isolated from injured muscles (Figure 9, D and E). The ability of Mrep to induce osteogenic differentiation of FAPs was diminished by activin A–neutralizing antibody treatment. These results suggest that Mrep triggers the development of HO through activin A production when ACVR1 is aberrantly activated.

Previously, clodronate treatment was reported to reduce HO in a FOP model (44). We further determined the pathological importance of Mrep-derived activin A in HO in vivo by evaluating the *Acvr1*^Q207D^-induced HO in macrophage-specific, activin A–deficient (*Inhba*^fl/fl^ LysM-Cre) mice. Deleting activin A in macrophages significantly reduced HO (Figure 9, F and G), indicating that Mrep-induced activin A is essential for HO in FOP. Mrep expression of activin A is dependent on TLR4-mediated signaling (Figure 6, D and E). TAK-242, a small molecule compound that inhibits TLR4 signal transduction (45), significantly inhibited activin A expression in muscles at 1 dpi (Figure 9H). The TAK-242 treatment reduced the volume of HO to a level similar to that of macrophage-depleted mice (Figure 9, I–K). Collectively, these results demonstrate that inhibition of activin A production from Mrep by TAK-242 could be a potential therapeutic approach for treating FOP.

## Discussion

In this study, we discovered that the unique macrophage subset Mrep, characterized by Ly6C^hi^CX3CR1^lo^PDPN^+^CD9^+^, exerts dual functions during muscle regeneration. Mrep has an indispensable role in muscle regeneration but can also cause muscle degeneration through the induction of HO under pathologic conditions in which ACVR1-mediated BMP signaling activity is aberrantly augmented. The molecular mechanism linking the opposing Mrep functions is reliant on its production of activin A stimulated by the TLR4/TRIF/TBK1/IRF3/7 signaling pathway upon muscle injury. Mrep accumulated at the injury site to promote muscle regeneration and, in turn, stimulated aberrant osteogenic differentiation of FAPs if ACVR1 had a mutation, such as R206H, by transducing BMP signaling through activin A binding. These findings provide important insights into the molecular mechanisms underlying muscle regeneration and show a possibility of therapeutic intervention in HO in FOP patients by targeting the upstream signaling of activin A production.

According to scRNA-seq analysis, Mrep also highly expresses certain genes known to promote muscle regeneration, such as *Tnf*, *Il15*, and *Hgf* (20, 33, 46) (Supplemental Figure 5G), in addition to activin A. Therefore, it is possible that Mrep promotes muscle regeneration through the orchestrated actions of these factors. Analysis of human muscle scRNA-seq data revealed a macrophage subcluster expressing activin A. Although this cluster did not express the full set of Mrep surface markers (PDPN, CD9, and IL7R), such differences may reflect species-specific variation. In addition, the human muscle scRNA-seq data were derived from surgically discarded tissues of otherwise healthy donors and therefore do not represent postinjury muscle, limiting direct comparison with the mouse injury model. Nevertheless, the presence of a subset of human macrophages that expressed activin A supports our observations in mice and suggests a potential role in human muscle repair, warranting future investigation. Moreover, GWAS using human muscle samples revealed that SNPs in certain genes, including *TLR4* and *ACVR2B* (a component of the receptor complex for activin A), showed an association with the loss of skeletal muscle mass in cancer patients (47), suggesting that the identified macrophage subset and pathway could also be involved in muscle regeneration in a human context.

Activin A belongs to the TGF-β superfamily and regulates a wide variety of cellular events, including proliferation, stem cell differentiation, apoptosis, and cancer metastasis, but its function in muscle regeneration has been controversial. We showed that activin A accelerates the proliferation of MuSCs and subsequent myotube formation (Figure 2, A–E). Consistent with our results, previous studies have shown that activin A facilitates the proliferation of C2C12 myoblasts (29). However, prolonged inhibition of activin A with a neutralizing antibody has been shown to promote muscle growth in both muscle hypertrophy and cardiotoxin-induced muscle injury models (27, 28). This may be attributed to the source-dependent effects of activin A on muscle regeneration. Muscle regeneration is a sequential process involving the activation and proliferation of MuSCs and myoblasts, the differentiation of myoblasts into myofibers, and the maturation of new myofibers. Activin A is expressed throughout this process, but its primary source changes: in the early phase, where the activation and proliferation of MuSCs and myoblasts occur, activin A is predominantly derived from macrophages and promotes myoblast proliferation, as presented in this study. In contrast, during the later phase, stromal cells become the main source of activin A, influencing the fusion of myofibers. Therefore, the enhanced myogenesis observed with long-term inhibition of activin A may result from increased differentiation or fusion, which compensates for the reduced proliferation driven by macrophage-derived activin A.

As a therapeutic target, enhancing activin A expression for muscle repair may raise concerns beyond FOP. In the absence of the *ACVR1* mutations, activin A does not activate osteogenic signaling, but instead competes with BMPs for receptor binding (48, 49), suggesting that HO as seen in FOP patients would be unlikely to occur in the broader population. However, it is important to acknowledge that muscle injury involves a complex interplay of various factors and cell types, including mesenchymal stem cells, which could potentially alter ACVR1 signaling or cellular responsiveness to activin A. Further studies are required to define how activin A can be harnessed safely and effectively to promote muscle regeneration.

We showed that Mrep comprises a muscle-repairing macrophage subset that induces the osteogenic differentiation of *Acvr1^Q207D^*-FAPs through the production of activin A. It has been shown that mast cell–deficient c-Kit^W-sh/W-sh^ mice exhibit a decrease in HO in a genetically induced HO model (44), but mast cells were barely detectable in the muscle injury site in our model. c-Kit is also expressed on germ cells, hematopoietic stem cells, and early hematopoietic progenitors (50), and macrophage infiltration has been shown to be reduced in c-Kit^W-sh/W-sh^ mice (51). Therefore, the contribution of mast cells to HO should be carefully investigated in future studies.

At present, there is no effective treatment for either trauma-induced or genetic HO, including that which occurs in FOP. Several compounds, such as palovarotene (RARγ agonist), garetosmab (activin A–neutralizing antibody) (52), saracatinib (ACVR1 inhibitor) (53), and rapamycin, have emerged as potential therapeutic agents for FOP. This study presents a promising strategy for the treatment of FOP by targeting TLR signaling and activin A production. A drug repurposing of TAK-242, which was originally developed to treat sepsis, is a promising approach in this regard. In summary, we have discovered a unique macrophage subset that functions as a sort of double-edged sword, promoting muscle regeneration physiologically on the one hand, while exacerbating disease progression on the other.

## Methods

### Sex as a biological variable.

Our study examined male and female animals, and similar findings are reported for both sexes.

### Mice.

C57BL6/J mice were purchased from CLEA Japan. CAGGCreER (CreER) (stock 004682), LysM-Cre (stock 004781), *Rosa26*-DTR (iDTR) (stock 007900), *Ccr2*^–/–^ (stock 004999), and CD45.1 (stock 002014) mice were purchased from The Jackson Laboratory. *Acvr1*^Q207D^ (CAG-Z-ACVR1^Q207D^-IRES-EGFP) transgenic mice, *Tlr4^–/–^,* *Myd88^–/–^*, *Ticam1^–/–^*, *Irf3^–/–^, Irf7^–/–^*, and *Inhba*^fl/fl^ mice were described previously (54–60). All the mice were maintained on a C57BL/6 background and kept under specific pathogen-free conditions. Sex-matched mice between 6 and 8 weeks of age were used for each experiment, unless otherwise indicated.

### Muscle injury.

Mice were anesthetized using isoflurane (Pfizer). Following an incision of the skin over the hind limb muscles, the hamstrings were transected at 2 sites, approximately 3 mm in length, using surgical scissors, as these muscles provide good surgical accessibility and reproducibility. The skin was then closed with 8-0 surgical sutures (Johnson & Johnson). In most experiments, the hamstrings were injured as described above. However, in certain experimental settings, the forelimb or gastrocnemius muscles were selected for injury as required. For the induction of muscle injury in LysM-Cre *Inhba*^fl/fl^ *Acvr1*^Q207D^ mice, 100 μL of 10 μM cardiotoxin (Sigma-Aldrich) in PBS was injected into the gastrocnemius muscle.

### In vivo treatment.

All reagents were administered intraperitoneally unless otherwise specified. For macrophage depletion, mice were administered 150 μL clodronate-encapsulated liposomes (clodronate) or control liposomes (FormuMax) 2 days before the muscle injury. For the long-term macrophage depletion in assessing the HO, 100 μL of clodronate or control liposomes was injected every 3 days after the first dose. For neutrophil depletion, 0.25 mg anti-Ly6G (1A8; BioXcell) or rat IgG2a isotype control (2A3; BioXcell) was injected into the mice 1 day before muscle injury. The efficiencies of macrophages and neutrophil depletion were confirmed by flow cytometry. For assessing the activin A expression in muscle at 1 dpi, mice were administered a single dose of 10 mg/kg of the following inhibitors on the day before muscle injury: TAK-242 (TLR4 inhibitor; Cayman), HMGB1 inhibitor (glycyrrhizin; Sigma-Aldrich), S100A8/A9 inhibitor (paquinimod; Sigma-Aldrich), TBK1 inhibitor (MRT67307; Sigma-Aldrich), and NF-kB inhibitor (caffeic acid phenethyl ester; Tocris Bioscience). To assess HO, mice were administered 10 mg/kg TAK-242 once daily for 5 days, starting the day before muscle injury. To inhibit ACVR1 kinase activity, mice were treated with 10 mg/kg LDN-193189. The administration procedures were conducted daily from the day of muscle injury until sacrifice for 28 days. For all the inhibitors, an equivalent volume of the corresponding solvent was administered on the same schedule as a control.

### Isolation of cells from muscles.

After euthanizing the mice, an incision was made in the skin of the hind limb to expose the muscles. The hind limb muscles were excised, and any attached fat and lymph nodes were removed. The muscles were then minced into small fragments using scissors. These fragments were subsequently digested in DMEM (Nacalai Tesque) containing 2 mg/mL Collagenase Type 2 (Worthington Biochemical). The digested muscle tissue was then centrifuged in 40% Percoll (GE Healthcare) to remove debris. The cell pellet was resuspended and filtered through a 70 μm cell strainer (Corning) for use in subsequent experiments.

### Flow cytometry analysis and antibodies.

Cell surface marker analyses and cell sorting were carried out on a FACS Fortessa (BD Biosciences) and a FACS Aria III (BD Biosciences). To prevent the unexpected binding of antibodies, cells were incubated with an Fc blocker (anti-mouse CD16/CD32; clone 2.4G2) before cell staining. Cells were stained with a mixture of the antibodies at a final concentration of 0.5 to approximately 1 μg/mL for 40 minutes at 4°C. The antibodies used for flow cytometry analysis were as follows: PE/Cy7 and APC/Cy7 anti-mouse CD3ɛ (clone 145-2C11); PerCP/Cy5.5 and PE/Cy7 anti-mouse CD9 (clone MZ3); PE/Cy7 anti-mouse CD19 (clone 1D3); Pacific blue and APC/Cy7 anti-mouse CD11b (clone M1/70); PE/Cy7 anti-mouse CD31 (clone 390); Pacific blue and Brilliant Violet 510 anti-mouse CD45 (clone 30-F11); FITC and PE/Cy7 anti-mouse CD45.1 (clone A20); Brilliant Violet 510 and Horizon V500 anti-mouse CD45.2 (clone 104); FITC anti-mouse CD106 (clone 429); PE anti-mouse CD127 (IL-7Rα; clone A7R34); APC and PE anti-mouse CD140a (PDGFRα; clone APA5); Pacific blue and PerCP/Cy5.5 anti-mouse CX3CR1 (clone 2A9-1); FITC, PE, and PE/Cy7 anti-mouse Ly6C (clone HK1.4); FITC and APC anti-mouse Ly6G (clone 1A8); PE anti-mouse F4/80 (clone BM8); Pacific blue anti-mouse NK1.1 (clone PK136); APC anti-mouse TCRβ (clone H57-597); PE anti-mouse TCRλ/δ (clone GL3); PerCP/Cy5.5 anti-mouse I-A/I-E (clone M5/114.15.2); PE and PerCP/Cy5.5 anti-mouse TER-119 (clone TER-119); APC and APC/Cy7 anti-mouse PDPN (clone NC-08); and APC/Cy7 and Pacific blue anti-mouse Sca-1 (clone D7). All the antibodies were purchased from BioLegend.

### MDM and Mrep transition analysis.

For tracing MDM transition, Ly6C^hi^CX3CR1^lo^ MDMs were sorted from muscles of CD45.2 mice on 1 dpi using a FACS Aria III. A total of 5 × 10^5^ cells, suspended in 50 μL PBS, were injected intramuscularly into the hamstrings of CD45.1 recipient mice. Flow cytometry was performed 3 days after the transfer to analyze the transitioned Ly6C^hi^CX3CR1^lo^ MDMs. For tracing Mrep transition, Mrep cells were sorted from muscles of CD45.1/CD45.2 heterozygous mice on 1 dpi using a FACS Aria III. A total of 5 × 10^5^ cells, suspended in 50 μL PBS, were injected intramuscularly into the hamstrings of CD45.2 recipient mice. Flow cytometry was performed 3 days after the transfer to analyze the transitioned CD45.1^+^CD45.2^+^ Mrep cells.

### RNA extraction and RT-qPCR.

Total RNA was extracted from muscle tissues or cell cultures using a Maxwell 16 LEV simplyRNA Tissue Kit (Promega) according to the manufacturer’s instructions. cDNA was synthesized using SuperScript III reverse transcriptase (Invitrogen). qPCR was performed on a LightCycler 480 II (Roche Applied Science) using SYBR Green Real-time PCR Master Mix (Toyobo) according to the manufacturer’s instructions. The primer sequences were as follows: *Gapdh*, 5′-ACCCAGAAGACTGTGGATGG-3′ and 5′-CACATTGGGGGTAGGAACAC-3′; *Inhba*, 5′-AACCACTACCGCATGAGGGG-3′ and 5′-TTTCTCTGGGACCTGGCGAC-3′; *Myog*, 5′-AACCAGCGGCTGCCTAAAGT-3′ and 5′-GGGACCGAACTCCAGTGCAT-3′. Melting curve analysis was performed to ensure the specificity of the amplification products.

### Bulk RNA-seq analysis.

Total RNA was isolated from uninjured hamstring muscles (*n* = 2), hamstring muscles at 1 dpi (vehicle, *n* = 2; clodronate, *n* = 3), and hamstring muscles at 4 dpi (vehicle, *n* = 2; clodronate, *n* = 2). cDNA was synthesized and amplified using the SMART-Seq v4 Ultra Low Input RNA Kit (Clontech Laboratories). Sequencing was performed on an Ion Proton platform (Thermo Fisher Scientific). RNA-seq data were processed and analyzed using CLC Genomics Workbench v9.0. Differentially expressed genes (DEGs) were identified using the DESeq2 package in R. Volcano plots were generated using the EnhancedVolcano package in R. A gene list of soluble factors was created based on the Gene Ontology (GO) term for cytokine activity (GO:0005125).

### Primary myoblast culture.

Total cells were obtained from uninjured muscle as described previously. MuSCs were isolated as the TER119^–^CD45^–^CD31^–^PDGFRα^–^Sca-1^–^CD106^+^ fraction using a FACS Aria III sorter. For the myotube formation experiment, sorted MuSCs were cultured on Matrigel-coated (Corning) coverslips (Falcon) in myoblast proliferation medium (DMEM supplemented with 20% FBS [Bovogen Biologicals], 3% basic fibroblast growth factor [Novus], and 1% penicillin-streptomycin [pen/strep] [Gibco]), with or without 100 ng/mL activin A. On day 4, when the expanded myoblasts reached over 90% confluence, the medium was switched to myoblast differentiation medium (Ham’s F-12 [Fujifilm] supplemented with 2% horse serum [Sigma-Aldrich] and 1% pen/strep) with or without 100 ng/mL activin A to induce differentiation. For immunofluorescence staining, differentiated myoblasts were fixed using 4% paraformaldehyde (PFA) and permeabilized with 0.2% Triton X-100. Myotubes were visualized using an anti-mouse myosin heavy chain antibody (MyHC; clone MF20; R&D Systems), followed by corresponding Alexa Fluor 488 secondary antibody (catalog A-11029; Thermo Fisher Scientific). Myotube formation was assessed by calculating the average number of nuclei within MyHC^+^ myotubes and the percentage of myotubes containing 4 or more nuclei. For the proliferation assay, 1 × 10^5^ sorted MuSCs were grown in proliferation medium. The expanded myoblasts were reseeded in a 6-well plate at a density of 1 × 10^5^ cells per well, with or without 100 ng/mL activin A, and cultured for 1–3 days. Myoblasts were detached and counted on the indicated day. For the differentiation assay, proliferated myoblasts were reseeded at a density of 30,000 cells/cm^2^ and cultured in myoblast differentiation medium, with or without 100 ng/mL activin A, for 2 days.

### scRNA-seq analysis.

Muscle tissues were harvested from 3 mice at 1 dpi. Cells were isolated, pooled together, and subsequently loaded onto a 10x Genomics Chromium chip for scRNA-seq. Reverse transcription and library preparation were performed using the Chromium Single Cell 3′ v3 reagent following the 10x Genomics protocol. Sequencing was performed on 1 lane per sample of an Illumina HiSeq 4000 with the following configuration (read 1, 28 bp; i5 index, 0 bp; i7 index, 8 bp; read 2, 91 bp). Sequences were mapped to the mm10 transcriptome using the 10x Genomics Cell Ranger pipeline. scRNA-seq data were analyzed using the Seurat package (v3.0 or v5.3.0) in R (3.6.2 or 4.5.1). Primary filtering excluded genes expressed in fewer than 3 cells and cells expressing fewer than 200 genes. Cells with more than 15% mitochondrial reads or expressing over 6,000 genes were removed. Raw counts were log-normalized to 10,000 counts per cell using the LogNormalize method. The top 2,000 variable genes were identified using the “vst” method for subsequent analysis. Principal component analysis was performed, and the number of principal components used for clustering was determined by the elbow method and set to 15. Clustering resolution was optimized by testing sequential values from 0.1 to 1.0. A resolution of 0.5 yielded the best results and identified 10 clusters. Hierarchical clustering was conducted based on Euclidean distance using the BuildClusterTree function in Seurat. DEGs for each cluster were identified using the FindAllMarkers function. DEGs among MDM subclusters were identified using the FindMarkers function, where each subcluster was compared against the other 2 subclusters. Clustering within the MDM subset was performed using a resolution of 0.5. Pathway enrichment analysis was conducted using the ReactomePA package in R. Cluster-specific signaling pathways were explored via GSEA using the GSEABase package in R. Cell type annotation was performed manually based on well-established marker genes. For the cardiotoxin-induced muscle injury scRNA-seq datasets (GEO GSE113111) (61), clustering was performed at a resolution of 0.4 using the first 20 dimensions with the Seurat package in R. For the human muscle scRNA-seq dataset (GEO GSE143704), the Duchenne muscular dystrophy mouse model (*mdx^5cv^* mice) dataset (GEO GSE218201), and the hind limb ischemia muscle dataset (GEO GSE227075), clustering was conducted as previously described (35–37).

### Measurement of activin A in the culture supernatant.

Mrep (Ly6C^hi^CX3CR1^lo^PDPN^+^CD9^+^ MDMs) and non-Mrep (the remaining MDM population) were directly seeded after sorting and cultured in DMEM supplemented with 10% FBS and 1% pen/strep. The supernatants were collected on day 2. The concentration of activin A was measured using an ELISA kit (R&D Systems) following the manufacturer’s instructions.

### Mrep transfer analysis in LysM-Cre iDTR mice.

To induce macrophage depletion in LysM-Cre iDTR mice, 400 ng of DT (Sigma-Aldrich) was injected intraperitoneally for 3 consecutive days prior to muscle injury. On day 0, the hind limb muscles of the LysM-Cre iDTR mice were injured. Immediately after muscle injury, 1 × 10^5^ cells of Mrep (Ly6C^hi^CX3CR1^lo^PDPN^+^CD9^+^ MDMs) or non-Mrep (other MDMs) suspended in 10 μL PBS were injected intramuscularly into the injury sites of DT-treated LysM-Cre iDTR mice. An equal volume of PBS alone was injected intramuscularly as a control. At 1 and 3 dpi, mice received additional DT injections. At 4 dpi, the mice were sacrificed for analysis. Donor Mrep (Ly6C^hi^CX3CR1^lo^PDPN^+^CD9^+^ MDMs) cells were sorted from either WT, *Inhba*^fl/fl^, or LysM-Cre *Inhba*^fl/fl^ mice 1 day after muscle injury. Donor non-Mrep cells were sorted from WT mice 1 day after muscle injury.

### Histological analysis.

Hind limb muscles were harvested at the indicated time points, fixed in 4% PFA overnight at 4°C, and subsequently embedded in paraffin. A series of cross sections were sliced at a thickness of 6 μm, with 150 μm intervals between sections, covering the entire embedded muscles. H&E staining was carried out by staining with hematoxylin (Muto Pure Chemicals) for 3 minutes followed by eosin (Wako) for 1 minute. For immunofluorescence staining, antigen retrieval was performed by immersing sections in 10% HistoVT One solution at 90°C for 20 minutes (Nacalai Tesque), followed by incubation with Blocking One Histo (Nacalai Tesque) for 10 minutes to block nonspecific binding. To analyze activin A expression in muscle tissue, sections were stained with APC anti-F4/80 antibody (clone BM8; BioLegend), anti-mouse activin A antibody (catalog AF338; R&D Systems), and corresponding Alexa Fluor 488 secondary antibody (catalog A-11029; Thermo Fisher Scientific). For the evaluation of muscle regeneration, sections were stained with anti-mouse embryonic myosin heavy chain (eMyHC) (catalog 22287-1-AP; Proteintech) and corresponding Alexa Fluor 594 secondary antibody (catalog A-11012; Thermo Fisher Scientific) to visualize regenerating fibers. Necrotic fibers were visualized using Alexa Fluor 488–conjugated anti-mouse IgG (mIgG) (catalog A-11029; Thermo Fisher Scientific), and nuclei were stained with Hoechst 33342 (Thermo Fisher Scientific). Since myofibers become permeable during necrosis, passive uptake of IgG proteins makes necrotic fibers positive for mIgG. To confirm the expression of the *Acvr1*^Q207D^ construct by IHC staining, sections were stained with 200 ng/mL anti-GFP antibodies (catalog ab183734; abcam) and rabbit IgG isotype control (catalog 910801; BioLegend), followed by overnight incubation at 4°C. Sections were then treated with a biotinylated universal antibody (catalog PK-4000; Vector Vecstain) for 30 minutes and processed using the Vector Vecstain ABC kit according to the manufacturer’s instructions to label HRP. Peroxidase activity was visualized by incubating sections with SIGMAFAST DAB tablets (Sigma-Aldrich) for 3 minutes. For von Kossa staining, deparaffinized sections were immersed in a 5% silver nitrate solution and exposed to direct sunlight for 1 hour. Afterward, the sections were treated with 5% sodium thiosulfate for 5 minutes, followed by Kernechtrot staining for 1 minute. H&E, IHC, and von Kossa images were captured using the BZ-X800 all-in-one fluorescence microscope (Keyence), and immunofluorescence images were captured using the AX-R confocal microscope (Nikon).

### Quantification of muscle regeneration.

The section with the largest injured area, selected from serial sections with 150 μm intervals, was chosen for quantification, as previously described (62). Muscle fibers showing cytoplasmic basophilia and centrally located nuclei in H&E sections or eMyHC^+^ fibers in immunofluorescence sections were manually outlined across the entire muscle section. For sections containing more than 200 regenerating fibers, at least 200 fibers were outlined. Their CSA was calculated using ImageJ2 (63). The regeneration area ratio was calculated by dividing the sum of the CSA of eMyHC^+^ fibers in a section by the total injured area. Necrotic fiber density was determined by dividing the total number of mIgG^+^ fibers in a section by the total injured area. The grip strength test was performed at 7 dpi by measuring the maximal forelimb force exerted by the mouse on a grid connected to a force sensor. Three consecutive trials were conducted, and the maximal value was used for statistical analysis. Muscle mass was measured after dissection of the gastrocnemius at 14 dpi.

### In vitro culture of BMDMs.

Bone marrow cells were isolated from the femur and cultured in DMEM supplemented with 10% FBS, 1% pen/strep, and 10 ng/mL macrophage colony-stimulating factor (R&D Systems). On day 7, BMDMs were collected with 0.25% trypsin EDTA (Gibco) and reseeded for subsequent stimulation. BMDMs were stimulated with 100 ng/mL LPS (Sigma-Aldrich), recombinant S100A8/A9 (1 μg/mL) (BioLegend), recombinant HMGB1 (1 μg/mL) (R&D Systems), heparan sulfate (1 μg/mL) (Sigma-Aldrich), low molecular weight hyaluronan (1 μg/mL) (R&D Systems), or muscle lysates. Muscle lysates were obtained by homogenizing 50 mg muscles at 1 dpi in 1.2 mL DMEM using a homogenizer (Minilys; Bertin Technologies). A volume of 100 μL muscle lysates was added to the BMDM culture. For the inactivation of the proteins, muscle lysates were heated at 95°C for 5 minutes.

### Induction of HO in Acvr1^Q207D^ mice.

*Acvr1*^Q207D^ CreER mice were generated by crossing *Acvr1*^Q207D^ mice with CreER mice. The *Acvr1*^Q207D^ construct consisted of a CAG-Z-ACVR1^Q207D^-IRES-EGFP cassette, where the CAG promoter drives the expression of the mutant *Acvr1*^Q207D^ gene and EGFP, but only after Cre-mediated recombination, as the expression is initially blocked by a LoxP-LacZ-triple pA-LoxP (Z) cassette (54). To induce *Acvr1*^Q207D^ expression, tamoxifen (75 mg/kg) was administered intraperitoneally to *Acvr1*^Q207D^ CreER mice at postnatal day 10. The successful Cre-mediated recombination was confirmed by IHC for GFP expression. For the induction of Cre recombination in *Inhba*^fl/fl^ *Acvr1*^Q207D^ and *Inhba*^fl/fl^ LysM-Cre *Acvr1*^Q207D^ mice, the adenoviruses containing Cre were injected intramuscularly into the hind limb muscles.

### MicroCT.

Injured hind limbs were dissected and fixed in 4% PFA. Three-dimensional images of each sample were acquired using the ScanXmate-D090S105 scanner (Comscantecno Co. Ltd.) with 360° rotation around the vertical axis. The x-ray source was set to 90 kV and 200 μA. Microstructural images were reconstructed using cone beam CT express software (White Rabbit), and bone mineral density was calculated using TRI/3D Bon 64 software (Ratoc System Engineering).

### In vitro osteogenic differentiation of FAPs.

FAPs (TER119^–^CD45^–^CD31^–^PDGFRα^+^Sca-1^+^) were sorted from the injured muscles of WT and *Acvr1*^Q207D^ mice. Primary FAPs were expanded for 2 days in DMEM supplemented with 10% FBS and 1% pen/strep. The expression of *Acvr1*^Q207D^ was induced by infected cells with retroviruses containing Cre. Retroviruses were produced using the Platinum-E (Plat-E) cell line (Cell Biolabs) that had been transfected with either pMXs-GFP control vector or pMXs-Cre-GFP vector. FAPs were then cultured in osteogenic differentiation medium (DMEM supplemented with 10% FBS, 1% pen/strep, 50 μg/mL ascorbic acid, 10 nM dexamethasone, and 10 mM β-glycerophosphate) for 14 days. For cytokine stimulation, 100 ng/mL recombinant activin A (R&D Systems) or BMP2 (R&D Systems) was added to the osteogenic differentiation medium. The culture medium was renewed every 3 days. Alizarin red S staining was performed day 14 from the culture starting point. Cells were fixed with 4% PFA and stained with alizarin red S solution (0.02 g/mL alizarin red S in H_2_O, pH 4.2).

### Coculture of Acvr1^Q207D^-FAPs with macrophages.

The FAPs were prepared as described above. Mrep (Ly6C^hi^CX3CR1^lo^PDPN^+^CD9^+^) and all the remaining CD45^+^ cells (non-Mrep) were prepared from muscles of WT mice at 1 dpi. A total of 5 × 10^4^ FAPs were cocultured with either 1 × 10^5^ Mrep or 1 × 10^5^ non-Mrep in the osteogenic differentiation medium. For the inhibition of activin A activity, 1 mg/mL anti–activin A antibody (catalog AF338; R&D Systems) was added to the osteogenic differentiation medium. On day 14, alizarin red S staining was performed as described above.

### Statistics.

Statistical analyses were conducted using GraphPad Prism 10 or individual R packages as indicated. The specific statistical tests used to calculate *P* values are indicated in each figure legend. A *P* value < 0.05 was considered statistically significant. Normality of the data was assessed using the Shapiro-Wilk test, and statistical analyses were performed accordingly. No prospective power calculation was performed. Group sizes were determined based on previous studies using similar models and were sufficient to detect statistically significant differences.

### Study approval.

All animal experiments were performed with the approval of the institutional committee of The University of Tokyo and conducted in accordance with institutional guidelines.

### Data availability.

The RNA-seq and scRNA-seq data generated in this study are available at NCBI’s Sequence Read Archive (BioProject PRJNA1356552) and in the Single Cell Portal hosted by the Broad Institute (study ID: SCP3391). Values for all data points in graphs are reported in the Supporting Data Values file.

## Author contributions

WY performed most of the experiments, interpreted the results, and wrote the manuscript. KO conceptualized and designed the study, interpreted the results, and contributed to the manuscript preparation. AT and WP contributed to the manuscript preparation and data interpretation. TO established the mouse model of muscle injury–induced HO and interpreted the results. MMM, SA, SU, and KM established and provided genetically modified mice and contributed to data interpretation and discussion. YH provided LDN-193189 and contributed to data interpretation. TIK and RB contributed to data interpretation and discussion. YM established genetically modified mice and contributed to data interpretation, manuscript preparation, and discussion. HT directed the project and wrote the manuscript.

## Funding support

- Japan Agency for Medical Research and Development (AMED)–Core Research for Evolutional Science and Technology (JP23gm1210008 and JP26gm2110004 to HT; JP23gm1710003 to KO).
- AMED Practical Research Project for Rare/Intractable Diseases (JP18ek0109379 to AT).
- AMED Practical Research Project for Allergic Diseases and Immunology (JP23ek0410108 to HT).
- Japan Science and Technology Agency FOREST Program (JPMJFR205Z to KO).
- Japan Society for the Promotion of Science, Scientific Research S (21H05046 to HT) and Scientific Research B (25K02757 to KO).
- SECOM Science and Technology Foundation (to HT).
- Mitsui Sumitomo Insurance Welfare Foundation (to KO).
- The Hokkoku Cancer Foundation (to KO).
- Astellas Foundation for Research on Metabolic Disorders (to KO).
- The Japanese Society for Bone and Mineral Research Rising Stars Grant (to KO).
- Extramural Collaborative Research Grant of Cancer Research Institute, Kanazawa University (to KO).
- Daiichi Sankyo Foundation of Life Science (to KO).
- The Naito Foundation (to KO).
- Chugai Foundation for Innovative Drug Discovery Science (to KO).
- MEXT Promotion of Development of a Joint Usage/Research System Project: Coalition of Universities for Research Excellence (JPMXP1323015484 to KO).
- Japan Initiative for World-leading Vaccine Research and Development Centers (AMED) (JP223fa627001).

## Supplementary Material

Supplemental data

Supporting data values

## Version 1 03/02/2026

Electronic publication
