# Supplementary material for: Activin A secretion by muscle-repairing macrophages induces heterotopic ossification in mice
Source: J Clin Invest. 2026 Mar 2;136(5):e193797. doi: 10.1172/JCI193797 (PMC12948425; doi:10.1172/JCI193797)

Supplemental materials for

Activin A secretion by muscle-repairing macrophages induces heterotopic ossification in mice

Wenqiang Yin,[1] Kazuo Okamoto,[2,3,4]* Asuka Terashima,[2,5] Warunee Pluemsakunthai,[1] Takehito Ono,[1,6] Taku Ito-Kureha, [1] Shizuo Akira,[7] Yoshinobu Hashizume,[8] Roland Baron,[9] Satoshi Ueha,[10] Kouji Matsushima,[10] Martin M Matzuk,[11] Yuji Mishina,[12] Hiroshi Takayanagi,[1]*

This PDF file includes:

Supplemental Figure 1 to 9

Supplemental Figure 1. Analysis of immune cell population in injured muscle. (**A**) Representative flow cytometry plots showing the immune cell populations in the muscle of wild-type mice at 1 dpi and 4 dpi (n = 3 or 4 for each group). Immune cells are identified as CD45^{+}; neutrophils as CD11b^{+}Ly6G^{+}; CD11b^{+}Ly6G^{-}; αβT cells as TCRβ^{+}; γδT cells as TCRγδ^{+}; B cells as CD19^{+}I-A/I-E^{+}; I-A/I-E^{+} cells as CD19^{-}I-A/I-E^{+}; NK cells as CD3ε^{-}NK1.1^{+}; the remaining cells as other. (**B**) Quantitative analysis of (A) showing the frequency of indicated cells in immune cells (CD45^{+}). (**C** and **D**) Representative flow cytometry plots (**C**) and quantification (**D**) showing the frequency of CD11b^{+}Ly6G^{-} cells in the muscle of *Ccr2*$^{-/-}$ mice at 1 dpi. The p-values were calculated using two-way ANOVA with Bonferroni correction for multiple comparisons (**B**) and unpaired two-tailed t-test (**D**). A p-value < 0.05 is considered significant. Error bars denote the mean ± SEM, and symbols represent individual mice.

Supplemental Figure 2. The transition of Ly6C^{high}CX3CR1^{low} MDMs after muscle injury. (**A** and **B**) Representative flow cytometry plots (**A**) and quantification (**B**) showing the analysis of MDMs based on the expression of Ly6C and CX3CR1 and quantitative analysis showing the frequency of Ly6C^{high}CX3CR1^{low} (1 dpi vs. 4 dpi, p = 0.0002) and Ly6C^{low}CX3CR1^{high} (1 dpi vs. 4 dpi, p = 0.0005) within the MDM population. (**C**) Experimental timeline for tracing the phenotypic changes of MDMs. Ly6C^{high}CX3CR1^{low} MDMs were sorted from CD45.2 mice at 1 dpi and transferred via intramuscular injection into CD45.1 recipient mice. The transferred MDMs were analyzed three days post-transfer. (**D**) Representative flow cytometry plots illustrate the gating strategy for isolating Ly6C^{high}CX3CR1^{low} MDMs from CD45.2 mice at 1 dpi. (**E**) Representative flow cytometry plots show the transition of transferred Ly6C^{high}CX3CR1^{low} MDMs (CD45.2^{+}) into Ly6C^{low}CX3CR1^{high} MDMs in the muscles of recipient CD45.1 mice. Data are representative of three independent experiments with similar results. (**F**) RT-qPCR results showing *Inhba* expression in MDMs sorted from muscles of wild-type mice at 1 dpi and 4 dpi. (**G**) Immunofluorescence staining of muscle sections at 1 dpi. Muscle sections were stained for anti-mouse activin A (cyan), anti-mouse F4/80 (red), and nuclei with Hoechst (blue). The panel displays a wide-field view of the injured muscle area. The boxed region is magnified, highlighting a cell with surface expression of F4/80 and cytoplasmic expression of activin A. Scale bars: 100 μm (wide-filed view), 10 μm (magnified). The p-values were calculated using two-way ANOVA with Bonferroni correction for multiple comparisons (**B**) and one-way ANOVA with Tukey's multiple comparisons test (**F**). A p-value < 0.05 is considered significant. Error bars denote the mean ± SEM, and symbols represent individual mice (**F**).

Supplemental Figure 3. Characteristics of the three subclusters of MDMs in injured muscle revealed by scRNA-seq analysis. (**A**) Experimental timeline for scRNA-seq. Total cells (TER119^{-}) were isolated from the muscles of three individual mice at 1 dpi and pooled for analysis. (**B** and **C**) Pre-processing of scRNA-seq data. Scatter plot showing the percentage of mitochondrial genes (**B**), and the number of detected genes (**C**). Cells with 200 to 6000 detected genes, and less than 15% mitochondrial gene content were selected for downstream analyses. (**D–I**) Violin plots showing the expression levels of marker genes used for annotation of cell types. MDMs, *Itgam* (CD11b), *Adgre1* (F4/80) and *Ccr2* (**D**); Neutrophils, *Itgam* (CD11b), *S100a8*, *Csf3r* and *Cxcr2* (**E**); DCs, high level of MHC class II molecules (*H2-Aa*, *H2-DMb1*) and *Ciita* (**F**). T cells, *Cd3g* (**G**); B cells, *Cd79a* (**H**); Stromal cells markers, no expression of *Ptprc* (CD45) but *Ly6a* (Sca-1) (**I**). Stromal cells are a mixed cell population, including endothelial cells (*Cdh5*) and fibroblasts (*Col4a2*).

Supplemental Figure 4. Marker genes for each cluster identified by scRNA-seq analysis. (**A**) Heatmap of the top 10 marker genes that were differentially expressed in each cluster.

Supplemental Figure 5. scRNA-seq–based expression profiling of MDM subclusters. (**A** and **B**) Representative flow cytometry plots (**A**) and quantification (**B**) show the frequency and number of neutrophils (CD11b$^+$Ly6G$^+$) in muscles at 1 dpi from the mice treated with isotype IgG (n = 4) or anti-Ly6G antibody (n = 4). (**C** and **D**) Dot plots representing the gene expression of M1-like macrophage markers (*Irf7*, *Il1b*, *Ccr2*, *Cd86*, *Cd68*, *Stat1*, *Tnf* and *Ly6c2*) (**C**), and M2-like macrophage markers (*Maf*, *Klf4*, *Vegfa*, *Stat2*, *Apoe*, *Clec4a1*, *Mrc1*, *Cx3cr1*, *Tgfb1* and *Arg1*) (**D**) across the Ly6C^{high}MHCII$^+$, Ly6C^{high}PDPN$^+$, and Ly6C^{low} subsets. The color scale represents average gene expression (log-transformed), and the dot size indicates the percentage of cells expressing the respective gene. (**E** and **F**) Violin plots showing the expression levels of *Mrc1*, *C1qa*, and *C1qc* (**E**), and *Ckb* (**F**) across Ly6C^{high}MHCII$^+$, Ly6C^{high}PDPN$^+$, and Ly6C^{low} MDMs. (**G**) Dot plots showing the expression of genes associated with muscle regeneration. (**H**) UMAP visualization of MDM subclusters. MDMs were subsetted from the scRNA-seq dataset shown in Figure 3A. This analysis identified four transcriptional subclusters (0–3). (**I**) Heatmap showing the top 10 differentially expressed genes by adjusted p value across MDMs subclusters. (**J** and **K**) Dot plots showing relative *Inhba* expression (**J**) and the expression of genes (*Pdpn*, *Cd9*, *Il7r*) (**K**) across the four MDM subclusters. *Inhba* expression was distributed across subclusters 1 and 2, along the *Pdpn*, *Cd9*, and *Il7r* genes that were preferentially expressed in the Ly6C^{high}PDPN$^+$ subset. The p-values were calculated using unpaired two-tailed t-test. A p-value < 0.05 is considered significant. Error bars denote the mean ± SEM, and symbols represent individual mice.

Supplemental Figure 6. MDMs-derived activin A is essential for adequate muscle regeneration. (**A**) Representative flow cytometry plots show the transition of transferred Ly6C^{high}CX3CR1^{low}PDPN^{+}CD9^{+} MDMs (isolated from CD45.2CD45.1 mice on 1 dpi). Data are representative of four independent experiments with similar results. (**B**) Relative count of Ly6C^{high}CX3CR1^{low} MDMs in muscle at 1 dpi in mice treated with PBS (n = 8) or DT (n = 6). (**C** and **D**) Representative flowcytometry plots (**C**) and quantification (**D**) showed the frequency and relative count of MDM in *Inhba*$^{fl/fl}$ (n = 3) and LysM-Cre *Inhba*$^{fl/fl}$ (n = 3) mice. (**E**) Experimental timeline of the transfer experiment. DT (400 ng/mouse) was administered intraperitoneally on the indicated days to LysM-Cre iDTR mice to induce macrophage depletion. Mrep (Ly6C^{high}CX3CR1^{low}PDPN^{+}CD9^{+} MDMs) were isolated from muscle tissues of *Inhba*$^{fl/fl}$ and LysM-Cre *Inhba*$^{fl/fl}$ mice at 1 dpi. The isolated cells, suspended in 10 µl PBS, were then injected intramuscularly into the recipient LysM-Cre iDTR mice. n = 3 for each group. (**F**). H&E staining of muscle sections from recipient LysM-Cre iDTR mice in (**E**) at 4 dpi. The boxed area is shown at higher magnification (x4). Scale bar: 100 µm. (**G** and **H**) Quantification of regeneration showing the CSA distribution (**G**) and the mean CSA of regenerating fibers (**H**). The p-values were calculated using unpaired two-tailed t-test. A p-value < 0.05 is considered significant. Error bars denote the mean ± SEM, and symbols represent individual muscle fibers (**G**) or individual mice (**B**, **D** and **H**).

Supplemental Figure 7. Analysis of public muscle scRNA-seq datasets. (A) UMAP plot showing clustering of 7 identified monocytes/macrophages subclusters from cardiotoxin muscle scRNA-seq data (GSE113111). **(B–D)** Plots show the expression of *Lyz2* (**B**), *Inhba* (**C**), *Pdpn*, *Cd9*, and *Il7r* (**D**) across macrophage subclusters. Cluster 4 preferentially expressed *Inhba* and Mrep markers (*Pdpn*, *Cd9*, and *Il7r*). **(E)** UMAP plot showing clustering of 16 identified cell populations from human muscle scRNA-seq data (GSE143704). These include fibroblast, endothelial cells (ECs), smooth muscle cells (SMCs), muscle progenitors, B/T/NK cells, adipocytes, monocytes/macrophages, pericytes, mature skeletal muscle cells (MSMs), and myonuclei. (**F**) Subclustering analysis of monocytes/macrophages yielded five subclusters. Clusters 0 and 1 comprised the majority of macrophages compared with clusters 2–4. (**G**) Expression profiles across macrophage subclusters revealed distinct populations. *CD14* was detected in clusters 0 and 1, indicating its expression in both circulating monocytes and monocyte-derived macrophages. Cluster 0 showed high levels of *MRC1*, *FOLR2*, *DAB2*, and HLA class II genes (*HLA-DRA*, *HLA-DRB1*, *HLA-DPB1*), consistent with differentiated macrophages. Cluster 1 expressed *S100A8*, *S100A9*, *S100A12*, *LYZ*, *FCN1*, and *VCAN*, indicating inflammatory monocytes. (**H**) Dot plot showing the expression of *INHBA* across macrophage subclusters. Cluster 1 highly expressed activin A. (**I** and **J**) Dot plots showing *Inhba* expression across the indicated clusters in scRNA-seq datasets from the skeletal muscles of a Duchenne muscular dystrophy mouse model (mdx^{5cv}) (**I**) or from hindlimb ischemia (**J**).

Supplemental Figure 8. Analysis of MDM subsets in *Ticam1*$^{-/-}$ mice. (**A**) Representative flowcytometry plots show the subsets of MDMs (CD11b^{+}Ly6G^{-}) in wild-type (n = 6) and *Ticam1*$^{-/-}$ (n = 4) mice. (**B–D**) Quantification shows the percentage MDMs (CD11b^{+}Ly6G^{-}) in CD45^{+} cells (B), the percentage of Ly6C^{high}CX3CR1^{low} cells within the MDM population (**C**), and the percentage of Mrep cells within the Ly6C^{high}CX3CR1^{low} subset (**D**) in wild-type and *Ticam1*$^{-/-}$ mice. The p-values were calculated using unpaired two-tailed t-test. A p-value < 0.05 is considered significant. Error bars denote the mean ± SEM., and symbols represent individual mice.

Supplemental Figure 9. Establishment of muscle injury-induced heterotopic ossification mouse model. (**A**) Experimental timeline for inducing heterotopic ossification genetically in gHO mice. Tamoxifen was injected intraperitoneally at postnatal day 10 to induce Cre recombination, thereby activating *Acvr1*^{Q207D} expression. Muscle injury was induced on postnatal day 20. The ACVR1^{Q207D} construct consisted of a CAG-Z-ACVR1Q207D-IRES-EGFP cassette, where the CAG promoter drives the expression of the mutant *Acvr1*^{Q207D} gene and EGFP, but only after Cre-mediated recombination, as the expression is initially blocked by a LoxP-LacZ-triple pA-LoxP (Z) cassette (52). (**B**) Successful Cre recombination in the gHO mice confirmed by immunohistochemical staining of the GFP expression in muscle sections at day 10 post-tamoxifen injection. Scale bar, 100 mm. The representative data from three independent experiments with similar results are shown. (**C**) RT-qPCR results show the relative *Inhba* expression in the muscles from uninjured wild-type mice (n = 3), injured wild-type mice (n = 3) and gHO mice (n = 3) at 1 dpi. (**D**) RT-qPCR results show relative *Inhba* expression in the indicated macrophage populations sorted from the muscles of wild-type (n = 4) and gHO mice (n = 4) at 1 dpi. (**E**) Confirmation of HO lesions (black arrows) in gHO mice at 28 dpi by von Kossa staining. The boxed region in the main image is shown at higher magnification in the adjacent lower panel. Scale bar, 100 μm. Representative data from three independent experiments with similar results are shown. (**F**) Flow cytometry gating strategy for sorting FAPs (TER119^{-}CD45^{-}CD31^{-}PDGFRα^{+}Sca-1^{+}). MuSCs were identified within the non-FAPs population. (**G**) Alizarin Red S staining of FAPs and non-FAPs, sorted from wild-type or *Acvr1*^{Q207D} mice, cultured with or without activin A (100 ng/ml) stimulation for 14 days. Representative data from three independent experiments are shown. The p-values were calculated using one-way ANOVA with

Tukey's multiple comparisons test (**C**) and two-way ANOVA with Bonferroni correction for multiple comparisons (**D**). A p-value < 0.05 is considered significant. Error bars denote the mean ± SEM, and symbols represent individual mice (**C** and **D**).

Supplemental Figure 1

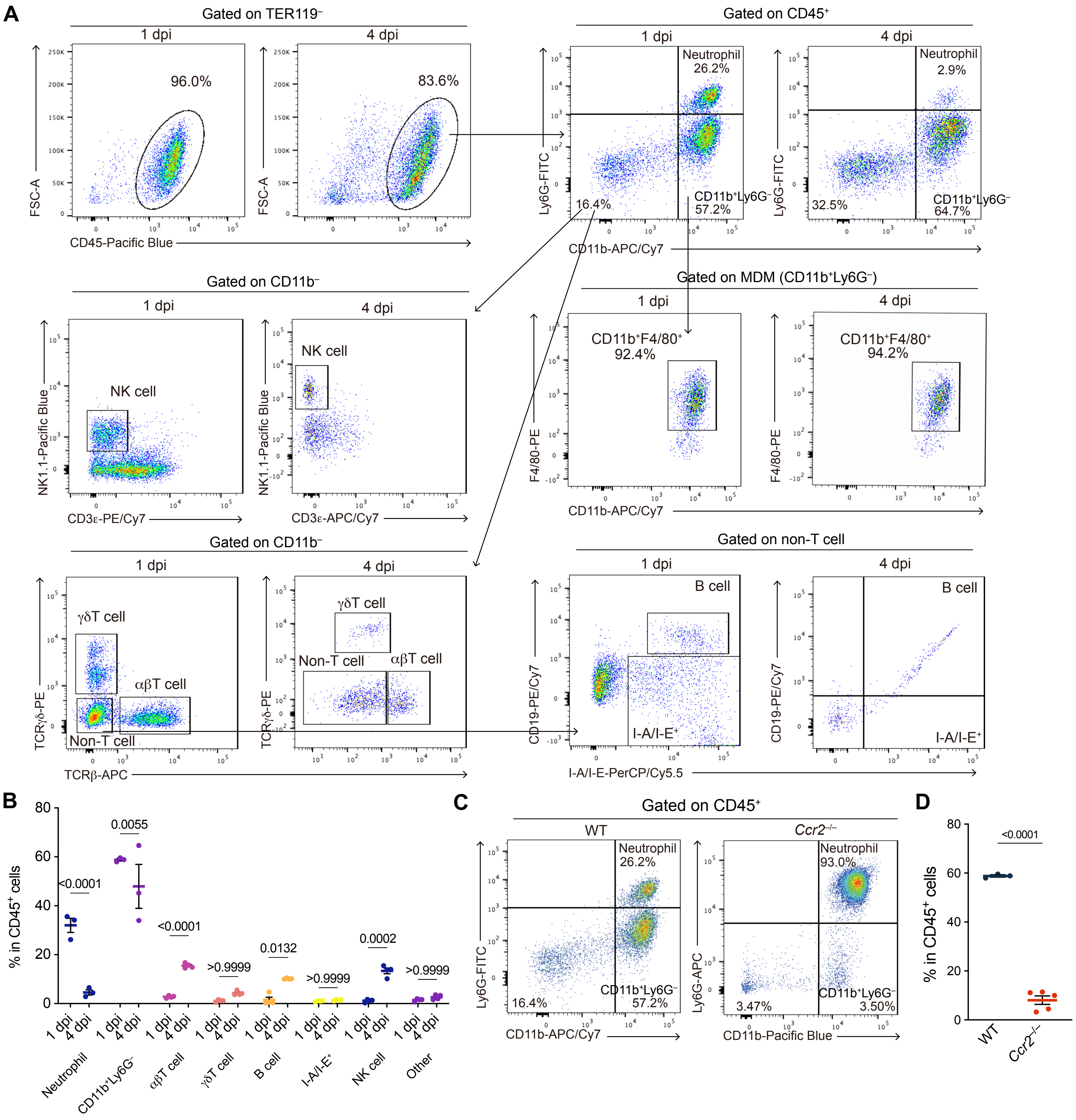

Supplemental Figure 2

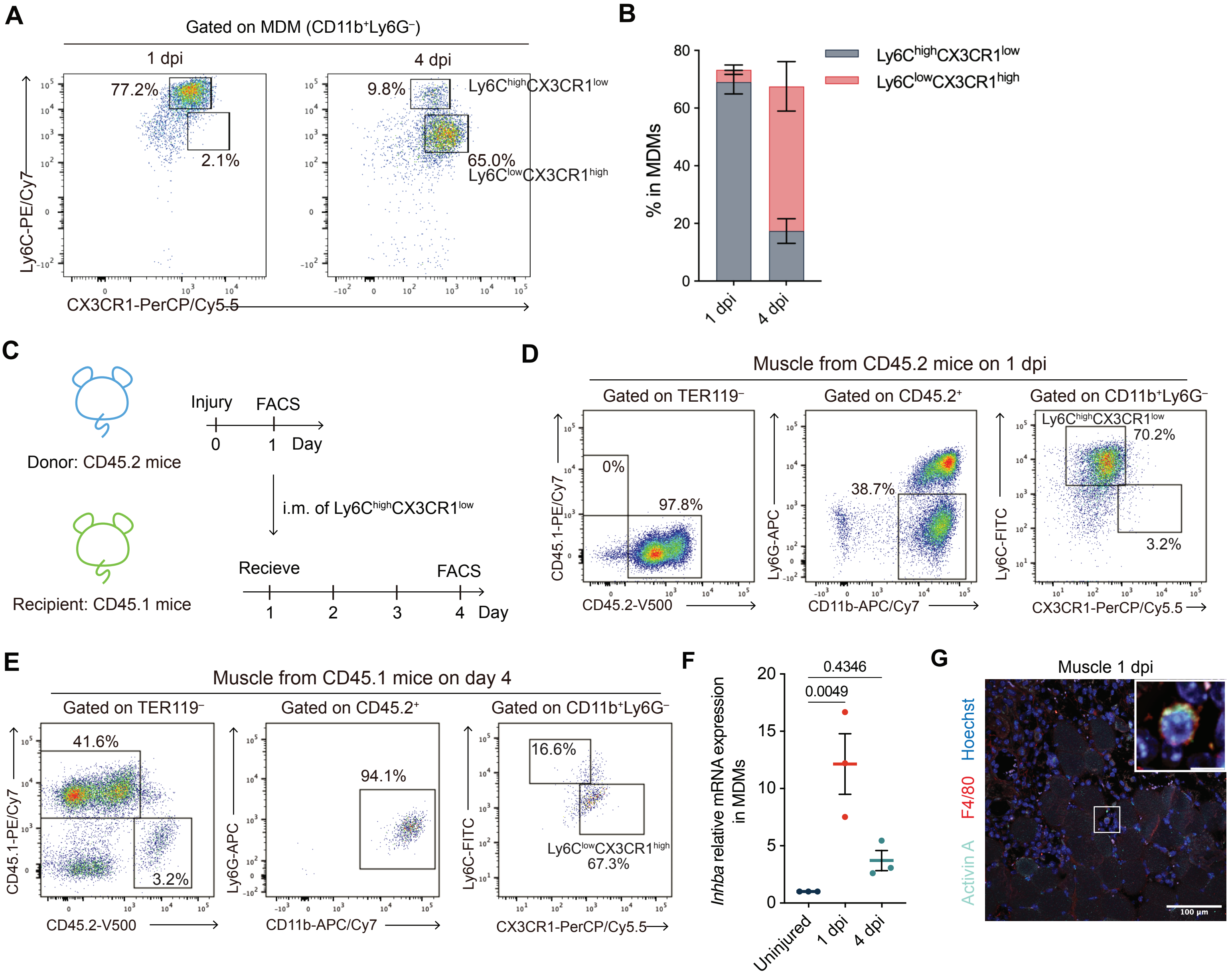

Supplemental Figure 3

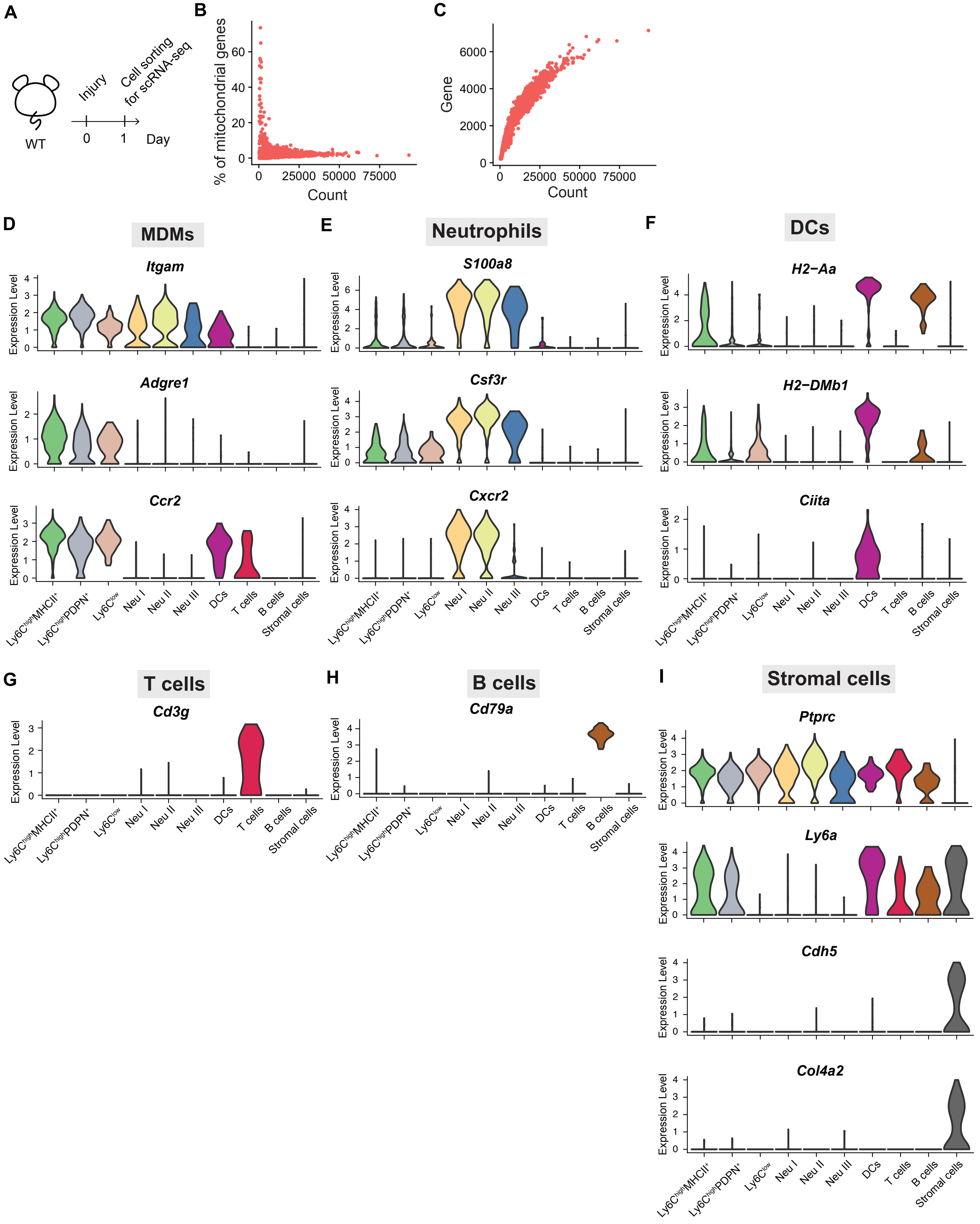

Supplemental Figure 4

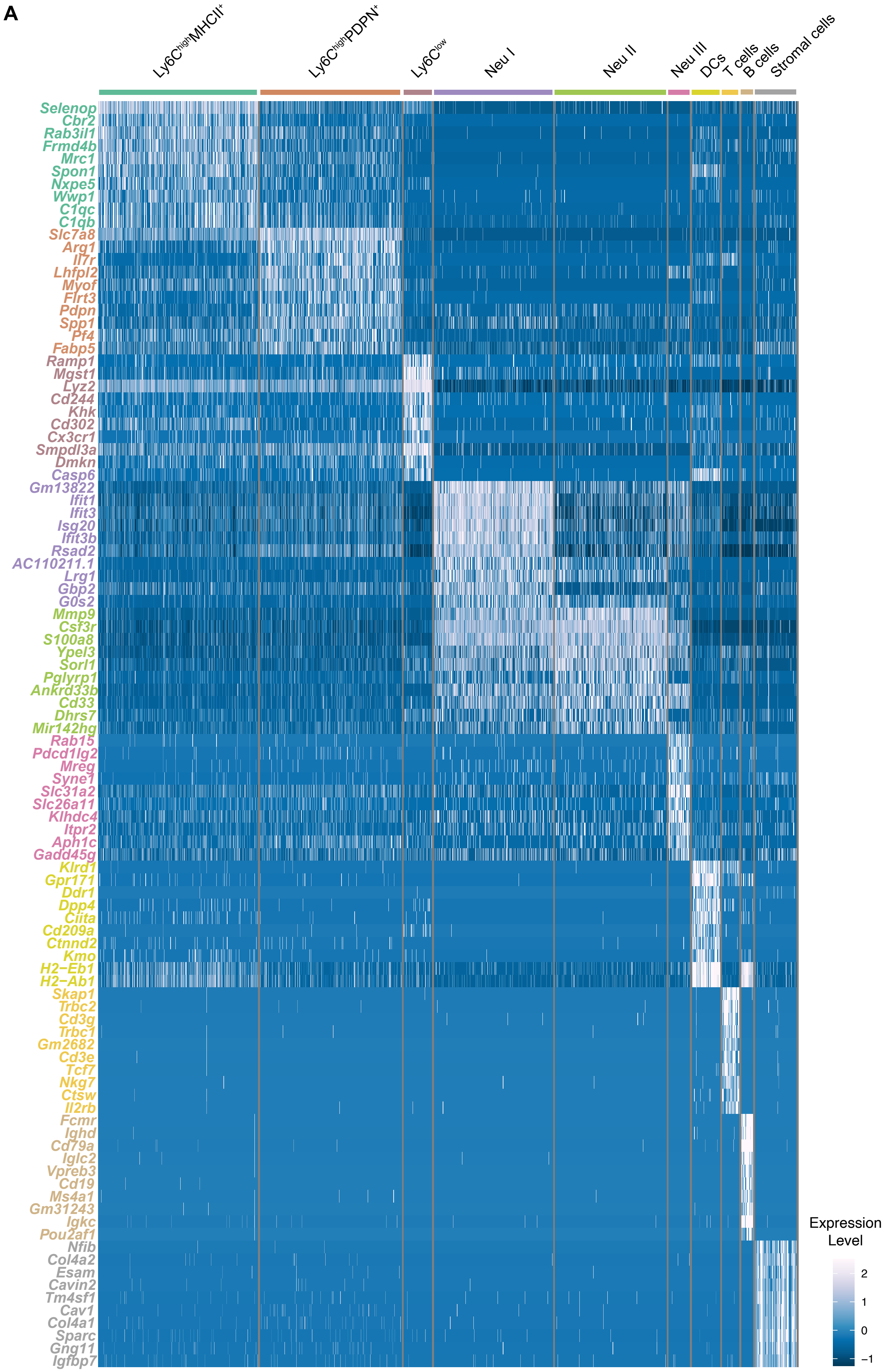

Supplemental Figure 5

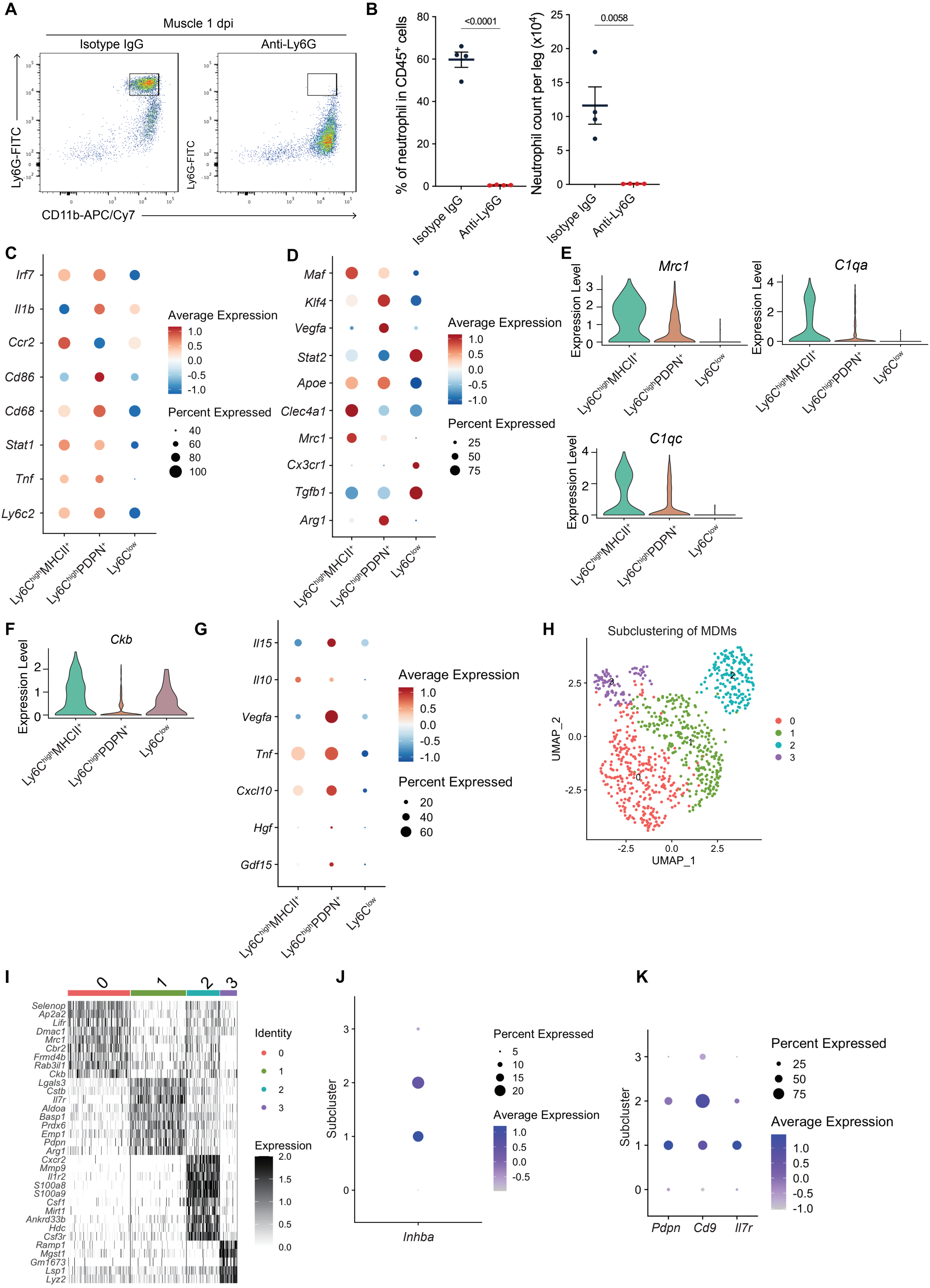

Supplemental Figure 6

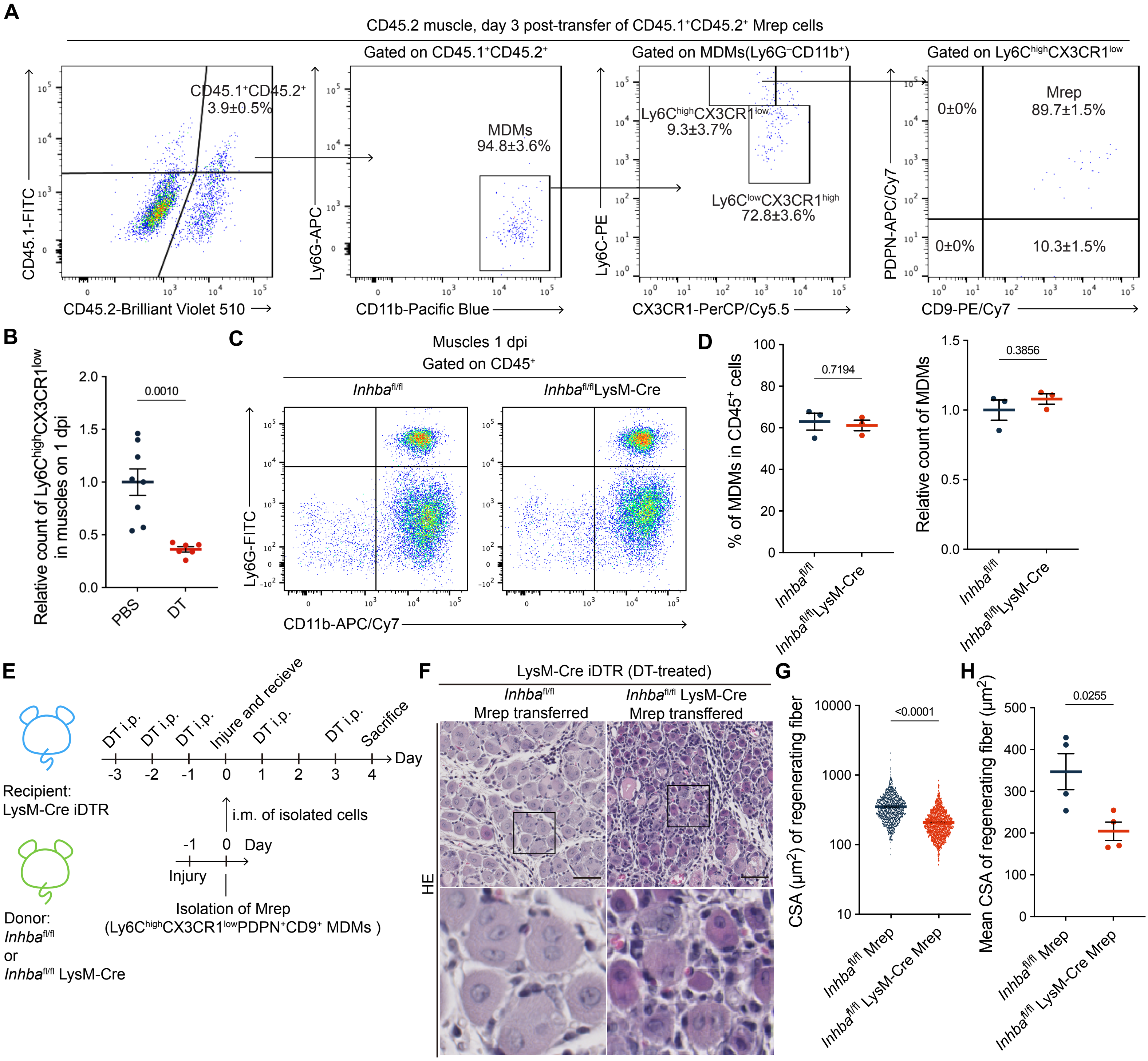

Supplemental Figure 7

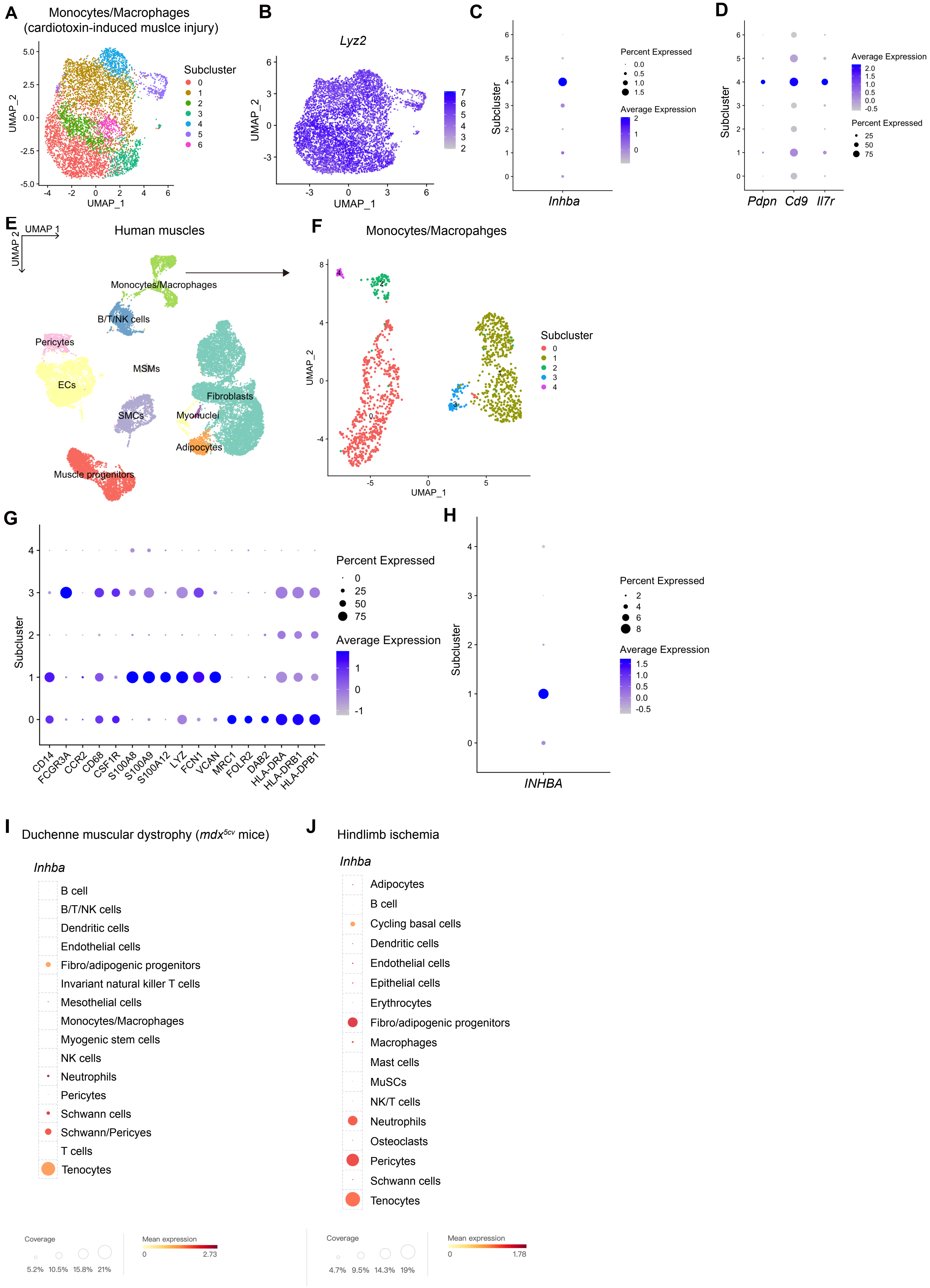

Supplemental Figure 8

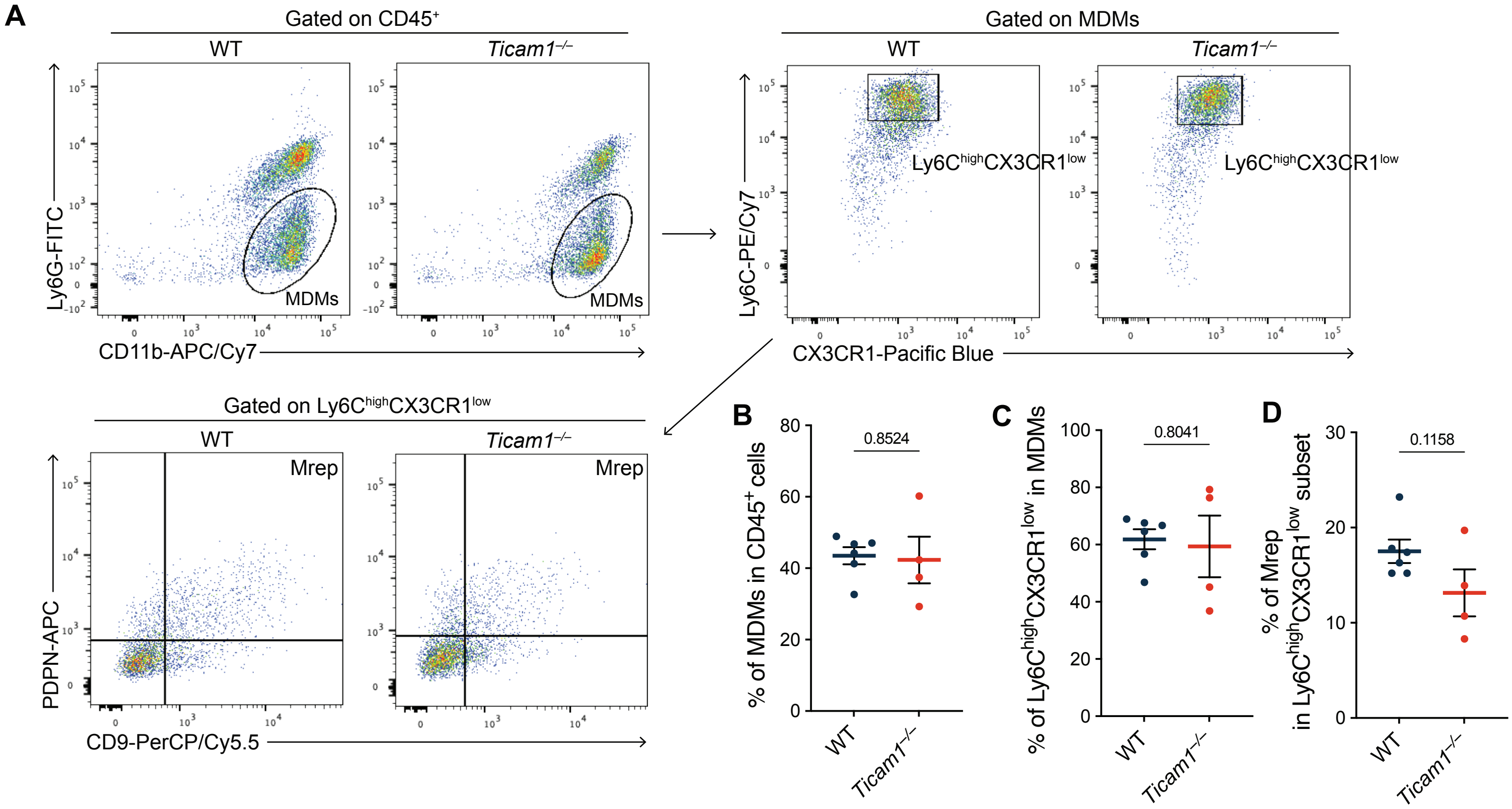

Supplemental Figure 9

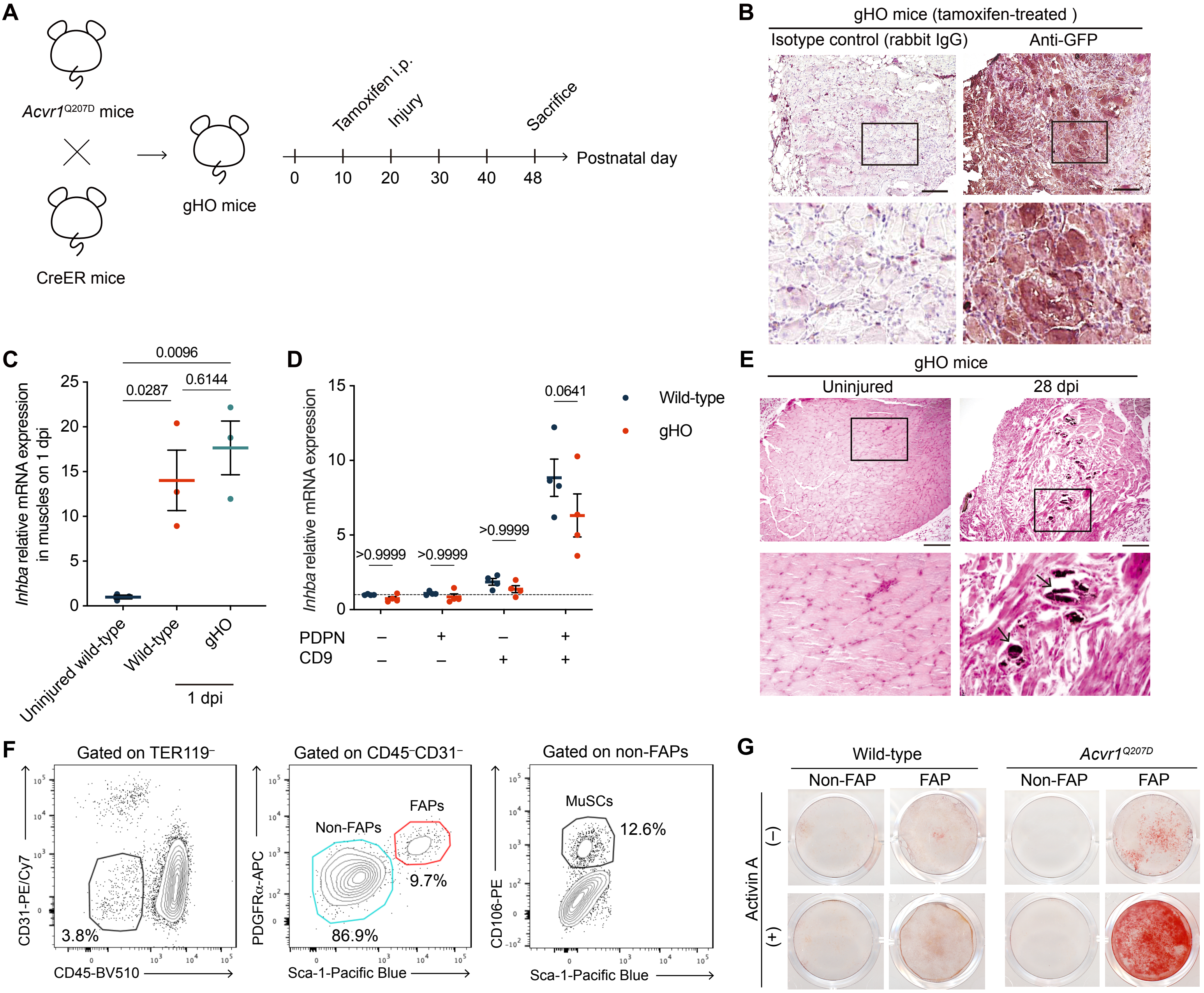

Supplement: Supplemental data [file jci-136-193797-s223.pdf]
